# Wnt5a-induced M2 polarization of tumor-associated macrophages via IL-10 promotes colorectal cancer progression

**DOI:** 10.1186/s12964-020-00557-2

**Published:** 2020-03-30

**Authors:** Qing Liu, Chaogang Yang, Shuyi Wang, Dongdong Shi, Chen Wei, Jialin Song, Xiaobin Lin, Rongzhang Dou, Jian Bai, Zhenxian Xiang, Sihao Huang, Keshu Liu, Bin Xiong

**Affiliations:** 1 Department of Gastrointestinal Surgery, Zhongnan Hospital of Wuhan University, No.169 Donghu Road, Wuchang District, Wuhan, 430071 China; 2 Department of Gastric and Colorectal Surgical Oncology, Zhongnan Hospital of Wuhan University, No.169 Donghu Road, Wuchang District, Wuhan, 430071 China; 3 Hubei Key Laboratory of Tumor Biological Behaviors, No.169 Donghu Road, Wuchang District, Wuhan, 430071 China; 4 Hubei Cancer Clinical Study Center, No.169 Donghu Road, Wuchang District, Wuhan, 430071 China

**Keywords:** Tumor-associated macrophages, Wnt5a, IL-10, M2 polarization, Colorectal cancer

## Abstract

**Background:**

Tumor-associated macrophages (TAMs) in the tumor microenvironment influence tumor initiation, invasion and metastasis. Several studies have shown that Wnt5a is mainly expressed in the tumor stroma, especially in TAMs. However, whether Wnt5a regulates the polarization and biological function of TAMs in colorectal cancer (CRC) is incompletely understood.

**Methods:**

Immunofluorescence staining was performed to detect CD68 and Wnt5a expression in colorectal tissues from patients (63 CRC specimens VS 20 normal tissues). RT-qPCR, flow cytometry, ELISA and inhibitors were carried out to explore the role of Wnt5a in the polarization of TAMs. Clone formation and transwell assays were performed to determine the effects of Wnt5a–treated macrophages on tumor proliferation, migration and invasion in vitro. Finally, a xenograft model was applied to confirm the effects of Wnt5a^+^ TAMs on CRC tumorigenesis.

**Results:**

We found that high Wnt5a^+^CD68^+^/CD68^+^ TAMs ratio was significantly associated with poor prognosis in CRC patients and Wnt5a^+^ TAM was an M2-like TAM subtype. Subsequently, we found that Wnt5a induced macrophages to secrete IL-10, which then acted as an autocrine cytokine to induce M2 polarization of these macrophages. IL-10 neutralizing antibody completely reversed the pro-M2 effect of Wnt5a. Mechanistically, the CaKMII-ERK1/2-STAT3 pathway was required for Wnt5a-mediated IL-10 expression in macrophages. Furthermore, Wnt5a-induced M2 macrophages promoted CRC cells proliferation, migration and invasion; knockdown of Wnt5a in TAMs significantly impaired the pro-tumor functions of TAMs.

**Conclusions:**

Our data indicate that Wnt5a could induce M2 polarization of TAMs by regulating CaKMII-ERK1/2-STAT3 pathway–mediated IL-10 secretion, ultimately promoting tumor growth and metastasis of CRC.

## Background

Metastasis is a major cause of cancer-related deaths [1]. Tumor microenvironment (TME) is closely correlated with tumor metastasis, growth and occurrence [2, 3]. TME is mainly composed of cancer cells, cancer-associated fibroblasts, immune cells and the non-cellular components [4]. As the most abundant immune cells in TME, tumor-associated macrophages (TAMs) can secrete various mediators such as cytokines or chemokines to suppress anti-tumor immune response, stimulate angiogenesis and finally enhance cancer cells proliferation, invasion, intravasation and dissemination for metastasis [5–7]. As TAMs exert an important role in cancer metastasis, our group studied the interaction between TAMs and cancer cells. We demonstrated that TAMs, especially M2 subtype, induced epithelial-mesenchymal transition (EMT) of cancer cells to promote circulating tumor cell-mediated colorectal cancer (CRC) metastasis [8]. Meanwhile, we also found that EMT-programmed cancer cells could remodel the tumor microenvironment and activate TAMs to M2 phenotype [9], thereby facilitating tumor progression and metastasis [10, 11]. Therefore, M2-like TAMs might be critical players in the crosstalk between cancer cells and their microenvironment [10, 12]. Although the cancer-promoting functions of M2-like TAMs are well defined, the potential mechanisms underlying M2-like TAMs polarization still need further exploration.

M2 polarization of TAMs is a multi-factor, multi-step and complex pathological process [13]. Recent studies have shown that Wnt signaling plays a major role in M2-like TAMs polarization [14, 15]. Wnt signaling pathway is generally considered to be divided into two types-canonical Wnt/β-catenin signaling and noncanonical Wnt signaling, which is mainly activated by 19 Wnt proteins identified in mammals [16].Wnt5a, a member of Wnt family, binds to the frizzled receptor and LRP5/6 co-receptors to regulate multiple signaling pathways [17], thereby altering TME by inducing adipocytes to dedifferentiate into fibroblast-like cells or mesenchymal cells [18]. Furthermore, Wnt5a was considered to participate in the differentiation of immune cell, including dendritic cells, B cells, as well as macrophages [19–21]. Previously, Wnt5a was mainly located in the TAMs of tumor stroma in breast cancer or basal cell carcinoma [22, 23]; Smith and colleagues consistently found that Wnt5a was mostly expressed in the tumor stroma of CRC, especially in TAMs [24]. These findings indicated that Wnt5a could be involved in the regulation of tumor immune microenvironment. Moreover, Bergenfelz et al. observed that Wnt5a was mainly located in CD163^+^ TAMs in breast cancer and could induce a tolerogenic phenotype of macrophages [25]. Based on the above studies and our previous results [8, 9], we speculated that Wnt5a might induce M2 polarization of TAMs to promote tumor development in CRC.

In the present study, we attempted to investigate the role and underlying mechanism of Wnt5a in the reprogramming of TAMs by a serial of clinical samples analyses, in vitro and in vivo experiments. Our results showed that high Wnt5a^+^CD68^+^/CD68^+^ TAMs ratio was significantly associated with poor prognosis in CRC patients. Wnt5a induced M2 polarization of TAMs by regulating CaKMII-ERK1/2-STAT3 pathway-mediated IL-10 secretion, thereby enhancing tumor growth, invasion and metastasis of CRC. These findings unveil a new insight into understanding the indirect role of Wnt5a in CRC progression.

## Materials and methods

### Patients and specimens

A total of 63 CRC samples and 20 normal colorectal tissues were collected from patients who were diagnosed as primary CRC by histopathology and underwent curative resection at the Zhongnan Hospital of Wuhan University (Wuhan, China) between January 2013 and April 2015. No patients had received adjuvant preoperative chemotherapy or radiotherapy. Moreover, all enrolled patients had available clinicopathological characteristics information and follow-up data. Formalin-fixed, paraffin-embedded tumor specimens were prepared after surgical resection. Informed consents were obtained from these patients. The study protocol was approved by the research ethics committee of Zhongnan Hospital of Wuhan University.

### Immunofluorescence staining

The Paraffin-embedded specimens were sectioned at 3 μm thickness. After dewaxing, antigen retrieval was made in the microwave of citrate buffer. The slices were then blocked with 3% BSA and stained overnight at 4 °C with the primary antibodies against human Wnt5a (1:100, Abcam), CD68 (1:100, Abcam), HLA-DR (1:100, Abcam), CD163 (1: 100, Abcam), IL-10 (1:100, Abcam). Subsequently, specimen sections were incubated with fluorochrome-conjugated secondary antibodies. Nucleus staining was performed using Diaminophenylindole (DAPI).

Before immunofluorescence staining, all samples were stained with hematoxylin and eosin (H&E) to ensure that all specimens contained representative CRC tissue. For each section, at least 6 fields of view (at 200× magnification) were used to calculate positive cells stained with indicated antibody. A quantitative analysis was performed to determine the numbers of infiltrating CD68^+^ and Wnt5a^+^CD68^+^ cells. All of the above-mentioned cells were counted manually. Only cells with macrophage-like morphology were included. Cell counting was carried out in a blinded manner by two research fellows familiar with histomorphology and immunofluorescence staining. If there was a disagreement, the analyses would be discussed with the authors.

### Cell culture and reagents

Human CRC cell lines (SW480, HT29, HCT116, DLD-1) were obtained from the cell bank of Chinese Academy of Sciences in Shanghai. Human monocyte cell line THP-1 was purchased from ATCC. Cells were cultured in RPMI 1640 medium (Hyclone) with 10% heat-inactivated fetal bovine serum (Wisent) and incubated in a humidified atmosphere with 5% CO2 at a constant temperature of 37 °C. Human recombinant Wnt5a and IL-10 protein were purchased from R&D Systems and were used at the final concentration of 500 ng/ml and 50 ng/ml respectively. Box 5 (Wnt5a inhibitor, Merck, USA), U0126 (p-ERK inhibitor, Merck, USA), S3I-201 (p-STAT3 inhibitor, Merck, USA), CK59 (p-CaMKII inhibitor, Merck, USA) were used at 100 μM, 20 ng/ml, 100 μM, 50 μM, respectively. Human neutralizing IL-10 antibody and isotype IgG were purchased from Biolegend, USA.

### Macrophage generation and differentiation

THP-1 cells were first treated with 100 ng/ml PMA (Sigma-Aldrich, USA) for 24 h to generate THP-1 macrophages (M0 macrophages), followed by induction with IL-4 (50 ng/ml, R&D Systems)/IL-13 (20 ng/ml, R&D Systems) or LPS (100 ng/ml, Absin)/ IFN-γ (20 ng/ml, Absin) for 48 h to differentiate into M2 or M1 macrophages. To mimic TAMs formation, M0 macrophages were co-cultured with colorectal cancer cells (HCT116 or DLD-1) in a 6-well transwell co-cultivation system (0.4 μm pore size, Corning, USA). After 48 h, the co-cultured macrophages were collected to obtain TAMs.

### Elisa

The level of Wnt5a and IL-10 was estimated in different culture media by an ELISA kit (R&D Systems) according to the manufacturer’s instructions. The presented value represented the average of 3 independent experiments.

### Flow cytometry

Macrophages were harvested from 6-well plates, washed twice with PBS, made into single-cell suspensions, and then incubated with antibodies (FITC Mouse anti-Human CD68, PE Mouse Anti-human CD163, APC Mouse anti-Human HLA-DR, all from BD Biosciences, USA) for 60 min at 4 °C. The stained cells were then washed twice and resuspended in the 200 μl flow buffer for analysis on a FACS Calibur flow cytometer (BD Biosciences, USA) using FlowJo software (FlowJo, USA).

### Reverse transcription-quantitative PCR (RT-qPCR)

Total RNAs were extracted using the Trizol Reagent (Invitrogen, USA) according to the manufacturer’s instructions. Then 2μg RNA was reverse transcribed to synthesize cDNA using the Primescript™ RT reagent Kit (Vazyme, Nanjing, China), followed by RT-qPCR using the SYBR-Green PCR Master Mix (Vazyme, Nanjing, China), which was performed on the BIO-RAD CFX96 Real-time PCR machine. The relative expression level of mRNA was computed by using 2^-ΔΔCt^ method. The sequences of all primers are listed in Table S2.

### Western blot

Total cellular proteins were extracted using RIPA lysis reagent (Aspen) containing a protease inhibitor cocktail (Aspen) with or without a phosphatase inhibitor (Bio-swamp). The protein concentration was measured by BCA protein assay reagent kit (Aspen) and mixed with 4 × SDS loading buffer (Bio-swamp) for denaturation in a 100 °C boiling water bath for 8 min. The proteins were then separated on 10% SDS-PAGE gels and transferred to PVDF membranes (Merck). The PVDF membranes were blocked with 5% skim milk for 90 min, washed three times, and then incubated with primary antibody at 4 °C overnight. Thereafter, the PVDF membranes were washed three times for 30 min and incubated with HRP-conjugated secondary antibodies for 90 min at room temperature. Finally, the membranes were again washed five times for 30 min, exposed to ECL developer (Aspen) and analyzed by Bio-Rad Image Lab software. The following primary antibodies used in this study were listed: Wnt5a (1:1000, Abcam), ERK1/2 (1:1000, Protein Tech), p-ERK1/2 (1:1000, CST), STAT3 (1:1000, Protein Tech), p-STAT3 (1:1000, CST), NF-κB p65 (1:1000, Protein Tech), p-NF-κB p65 (1:1000, CST), CaMKII (1:1000, Protein Tech), p-CaMKII (1:1000, CST), GAPDH (1:5000, Protein Tech), α-Tubulin (1:5000, Protein Tech).

### Cell transfection

Lentiviral-mediated shRNA interference was performed as described previously [26]. Wnt5a expression was knocked down in SW480 or TAMs by transduction of a lentiviral vector expressing a short hairpin RNA–Wnt5a shRNA (sh-Wnt5a) or negative control shRNA (sh-NC) (Genechem, China). Transfections were performed using X-treme GENE HP reagent (Roche, USA) according to the manufacturer’s instructions.

### CCK-8 and clone formation assay

Cell viability was estimated using a CCK-8 reagent kit (Vazyme, Nanjing, China). 5000 CRC cells were planted in 96-well plates and cultured for indicated time, followed by addition of CCK-8 solution. Then, the spectral absorbance in each well was quantified at 450 nm using a microplate reader (Molecular Devices, USA). For colony formation detection, co-cultured HCT116 or DLD-1 cells were washed, digested, counted and seeded with 500 cells per well in a 6-well plate. After 2 weeks, colonies were fixed with 4% paraformaldehyde and then stained with 0.5% crystal violet (Sigma). Each clone that had more than 50 cells was counted. All experiments were performed in triplicate.

### Transwell migration and invasion assay

The experiments were performed using a 24-well transwell system (8 μm pore size; Corning, USA). For the migration assay, co-cultured 5 × 10^4^ HCT116 or DLD-1 cells were suspended in 600 μl RPMI 1640 containing 1% FBS and seeded into the upper chamber, while 700 μl RPMI 1640 containing 10% FBS was added to the lower chamber. After incubation for 48 h, the chamber was fixed with 4% paraformaldehyde and then stained with 0.5% crystal violet. Cells in the upper chamber were removed with a cotton swab. Cells in 4 randomly microscopic fields (at × 100 magnification) were counted and photographed. For the invasion assay, the upper chamber was coated with Matrigel (BD Biosciences, USA) before inoculation. The remaining steps were the same as the migration assay. Each experiment was performed in triplicates.

### In vivo experiments

Twenty-four BALB/c female nude mice (6–8 weeks old and 20–25 g) were purchased from the Hubei Research Center of Laboratory Animals (Wuhan, China). All animal experiments were conducted strictly in compliance with the guidelines of the Institutional Animal Care and Use Committee of the Zhongnan Hospital of Wuhan University. Nude mice were randomly divided into 4 groups (*n* = 6 per group). HCT116 cells alone (5 × 10^5^), TAMs alone (1 × 10^5^), HCT116 cells (5 × 10^5^) + sh-NC TAMs (1 × 10^5^), HCT116 cells (5 × 10^5^) + sh-Wnt5a TAMs (1 × 10^5^) were separately mixed, resuspended with 200ul PBS, and then subcutaneously injected into the right flank of mice. After 10 days, the size of the tumors was measured with digital vernier calipers once a week. The volume of the tumors was calculated according to the formula: volume = (length × width^2^)/2. After 45 days of cells inoculation, 1 ml of blood per mouse was harvested by cardiac puncture into EDTA-containing tubes, and then nude mice were sacrificed. Tumor tissues were collected and measured for weight and volume, followed for further analysis by RT-qPCR assay or IHC staining. In addition, liver and lung tissues of mice were collected to further evaluate the metastatic burden by H&E staining.

### Immunohistochemistry

The sample sections were subjected to dewaxing, antigen retrieval, incubation with 3% hydrogen peroxide solution and 3% BSA blocking. Slides were subsequently incubated with primary antibodies against human Wnt5a (1:100, Abcam), CD163 (1:100, Abcam), IL-10 (1:100, Abcam), Ki67(1:500, Abcam) at 4 °C overnight, followed by incubation with HRP-labeled secondary antibodies. Immunoreactions were visualized using enzymatic Avidin-Biotin Complex (ABC)-diaminobenzidine (DAB).

### CTC enrichment

Circulating tumor cells (CTCs) were captured, isolated, and identified by a novel CTCBIOPSY method as reported by our previous study [8, 9, 27]. Briefly, 1 ml of blood of mice was first diluted with 4 ml of 0.9% physiological saline and then totally transferred to CTCBIOPSY tubes with an 8-μm diameter aperture membrane. The diluted blood samples were filtered by positive pressure through the membrane, where CTCs were captured. CTCs on the membrane were stained with the primary antibodies (CK, CD45, Hoechst, all from Abcam) and then counted under a microscope (BX51-Olympus, Japan).

### Statistics analysis

All results were presented as means ± SEM from at least three independent experiments. Statistical analyses were performed using SPSS statistical software (version 19.0, IBM, USA). Means of continuous outcome variables were appropriately tested with one-way analyses of variance or two-tailed Student’s t-test. The clinical feature correlation analysis was performed using chi-squared test. Kaplan–Meier curves were applied to survival analysis, and log-rank test was used to assess differences in different subgroups of patients. Univariate and multivariate Cox-regression analyses were performed to determine independent prognostic factors. Differences were considered statistically significant when *p* values < 0.05.

## Results

### The ratio of Wnt5a^+^CD68^+^/CD68^+^ TAMs is correlated with poor prognosis in CRC patients

**Fig. 1:**
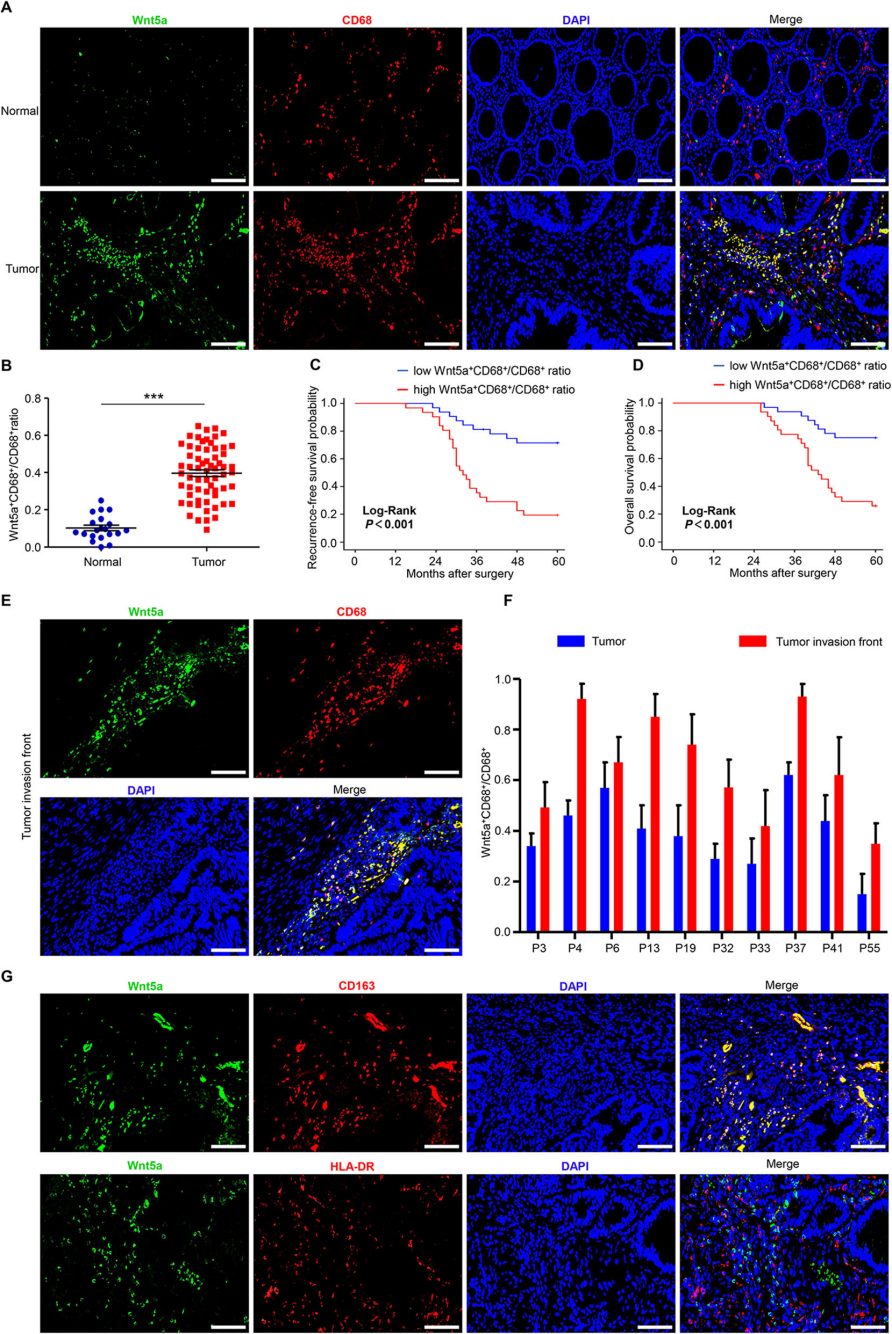
The ratio of Wnt5a^+^CD68^+^/CD68^+^ TAMs is correlated with poor prognosis in CRC patients. **a** Representative immunofluorescence staining images for Wnt5a (green), CD68 (red), DAPI (blue) in CRC samples. Bar = 100 μm. **b** Wnt5a^+^CD68^+^/CD68^+^ TAMs ratio was significantly elevated in primary human CRC tissues compared with normal colorectal tissues. Statistical analysis was conducted using one-way ANOVA. **c, d** Association of Wnt5a^+^CD68^+^/CD68^+^ TAMs ratio with recurrence-free survival and overall survival of CRC patients. **e** Representative immunofluorescence staining images for Wnt5a (green), CD68 (red), DAPI (blue) at tumor invasive front. Bar = 100 μm. **f** Wnt5a^+^CD68^+^/CD68^+^ TAMs ratio at tumor invasive front and tumor nest in 10 CRC samples. **g** Representative immunofluorescence photographs for co-localization staining of Wnt5a, M2 maker (CD163) and M1 maker (HLA-DR). Bar = 100 μm. Error bars, SEM. ***P < 0.001

In order to determine the relationship between Wnt5a and TAMs, we first detected the expression of Wnt5a and TAM marker (CD68) in 20 normal colorectal tissues and 63 CRC specimens. Immunofluorescence analysis revealed that Wnt5a expression was markedly elevated in CRC compared with normal tissue (Fig. 1a). Intriguingly, we found that Wnt5a was mainly expressed in the tumor stroma, especially in TAMs, with no or scarce expression in tumor nest (Fig. 1a, Fig. S1A). Moreover, we firstly observed that the number of Wnt5a^+^ TAMs was significantly increased in CRC samples compared to normal tissues (Fig. S1B). High Wnt5a^+^ TAMs expression was significantly correlated with LVI and TNM stage (Table S1). Further survival analysis showed that high Wnt5a^+^ TAMs expression was significantly associated with poor RFS and OS (Fig. S1 C and D), while CD68^+^ TAMs expression was insignificantly correlated with the prognosis of CRC patients (Fig. S1E and F).

We then estimated the significance of Wnt5a^+^CD68^+^/CD68^+^ TAMs ratio. We categorized all CRC patients into high and low Wnt5a^+^CD68^+^/CD68^+^ ratio groups using the median ratio value as a cutoff threshold. Similar to Wnt5a^+^ TAMs, Wnt5a^+^CD68^+^/CD68^+^ TAMs ratio was dramatically higher in CRC specimens than that in normal tissues (Fig. 1b). High Wnt5a^+^CD68^+^/CD68^+^ ratio was significantly correlated with LVI, TI, LNM and TNM stage (Table 1). Further survival analysis showed that a higher ratio of Wnt5a^+^CD68^+^/CD68^+^ TAMs was significantly associated with worse RFS and OS (Fig. 1c and d). Univariate and multivariate analyses revealed that only Wnt5a^+^CD68^+^/CD68^+^ TAMs ratio in the above-mentioned indicators was an independent prognostic factor associated with poor RFS and OS (Table 2). These results indicate that Wnt5a^+^ TAM is a poor prognostic indicator, and the ratio of Wnt5a^+^CD68^+^/CD68^+^ TAMs can serve as a marker for evaluating prognosis.

**Table 1:** Correlation between the ratio of Wnt5a^+^CD68^+^/CD68^+^ macrophages and clinicopathologic parameters (*n* = 63)

Parameters	n (%)	Wnt5a^+^CD68^+^/CD68^+^ ratio (n)		*χ*^2^ value	*P* value
		Low (*n* = 32)	High (*n* = 31)		
Gender				0.024	0.877
Male	38 (60.32)	19	19		
Female	25 (39.68)	13	12		
Age (years)				1.905	0.167
<60	29 (46.03)	12	17		
≥ 60	34 (53.97)	20	14		
Tumor site				0.013	0.910
Colon	35 (55.56)	18	17		
Rectum	28 (44.44)	14	14		
Tumor size (cm)				0.386	0.535
<5	41 (65.08)	22	19		
≥ 5	22 (34.92)	10	12		
Tumor grade				1.932	0.165
Poor	38 (60.32)	22	16		
Moderate/Well	25 (39.68)	10	15		
LVI				5.773	**0.016**
Absence	30 (47.62)	20	10		
Presence	33 (52.38)	12	21		
PNI				1.905	0.167
Absence	34 (53.97)	20	14		
Presence	29 (46.03)	12	17		
TI				6.719	**0.010**
T1–2	15 (23.81)	12	3		
T3–4	48 (76.19)	20	28		
LNM				7.014	**0.008**
N0–1	31 (49.21)	21	10		
N2–3	32 (50.79)	11	21		
TNM stage^#^				9.908	**0.002**
I/II	33 (52.38)	23	10		
III	30 (47.62)	9	21		
CEA (ng/ml)				0.128	0.721
<5	44 (69.84)	23	21		
≥ 5	19 (30.16)	9	10		

Notes: Bold indicates *P* < 0.05; #The 7th edition of the AJCC Cancer Staging Manual. Abbreviations: *LVI* Lymphovascular invasion; *PNI* Perineural invasion; *TI*, tumor invasion; *LNM* Lymph node metastasis; *TNM* Tumor-node-metastasis; *CEA* carcinoembryonic antigen; *CD68* Cluster of differentiation 68; *Wnt5a* Wingless-type MMTV integration site family, member 5a

**Table 2:** Univariate and multivariate analyses of clinicopathologic parameters associated with recurrence-free survival and overall survival

Parameters	Recurrence-free survival						Overall survival					
	Univariate analysis			Multivariate analysis			Univariate analysis			Multivariate analysis		
	HR	95% CI	*P*	HR	95% CI	*P*	HR	95% CI	*P*	HR	95% CI	*P*
Gender (Male vs. Female)	0.782	0.391–1.564	0.488				0.67	0.321–1.400	0.287			
Age (≥60 years vs. <60 years)	0.639	0.325–1.255	0.194				0.641	0.316–1.303	0.219			
Tumor site (Colon vs. Rectum)	1.015	0.515–2.001	0.965				1.418	0.700–2.876	0.332			
Tumor size (<5 cm vs. ≥ 5 cm)	1.255	0.627–2.512	0.521				1.474	0.721–3.016	0.288			
Tumor grade (Well vs Moderate vs Poor)	1.755	0.894–3.446	0.102				1.263	0.623–2.564	0.517			
LVI (Absence vs. Presence)	**2.134**	**1.051–4.333**	**0.036**	1.998	0.937–4.257	0.073	**2.51**	**1.178–5.350**	**0.017**	**2.336**	**1.040–5.251**	**0.040**
PI (Absence vs. Presence)	1.557	0.793–3.059	0.198				1.955	0.957–3.997	0.066			
TI (T1–2 vs. T3–4)	**2.288**	**1.299–4.029**	**0.004**	1.335	0.686–2.597	0.394	**1.926**	**1.094–3.390**	**0.023**	1.104	0.562–2.168	0.774
LNM (N0–1 vs. N2–3)	**1.464**	**1.070–2.002**	**0.017**	0.648	0.366–1.147	0.136	**1.387**	**1.001–1.921**	**0.049**	0.630	0.348–1.139	0.126
TNM stage^#^ (I vs. II vs. III)	**3.423**	**1.702–6.887**	**0.001**	**5.133**	**1.246–21.156**	**0.024**	**2.884**	**1.428–5.823**	**0.003**	**4.693**	**1.101–20.011**	**0.037**
CEA (≥ 5 ng/ml vs. <5 ng/ml)	0.791	0.377–1.657	0.533				0.529	0.227–1.231	0.14			
CD68^+^ macrophage (Low vs. High)	1.785	0.901–3.537	0.097				1.465	0.722–2.975	0.29			
Wnt5a^+^CD68^+^ macrophage (Low vs. High)	**2.204**	**1.088–4.465**	**0.028**	0.981	0.441–2.182	0.962	**2.184**	**1.045–4.563**	**0.038**	0.925	0.404–2.115	0.853
Wnt5a^+^CD68^+^/CD68^+^ macrophage (Low vs. High)	**4.596**	**2.126–9.935**	**< 0.001**	**2.698**	**1.110–6.559**	**0.029**	**4.347**	**1.934–9.772**	**< 0.001**	**2.809**	**1.107–7.128**	**0.030**

Notes: Bold indicates *P* < 0.05; #The 7th edition of the AJCC Cancer Staging Manual. Abbreviations: *LVI* Lymphovascular invasion; *PNI* Perineural invasion; *TI* Tumor invasion; *LNM* Lymph node metastasis; *TNM*, tumor-node-metastasis; *CEA* Carcinoembryonic antigen; *CD68* Cluster of differentiation 68; *Wnt5a* Wingless-type MMTV integration site family, member 5a

Furthermore, a higher Wnt5a^+^CD68^+^/CD68^+^ ratio was observed at the tumor invasive front (Fig. 1e and f), where there exists M2-like TAMs infiltration [8, 12]. So, we speculated that Wnt5a^+^ TAM might be an M2-like TAM subtype. Further immunofluorescence analysis showed that Wnt5a was mainly co-expressed with CD163 (M2 marker) but not with HLA-DR (M1 marker) (Fig. 1g).

### Wnt5a is mainly expressed in M2-like TAMs

**Fig. 2:**
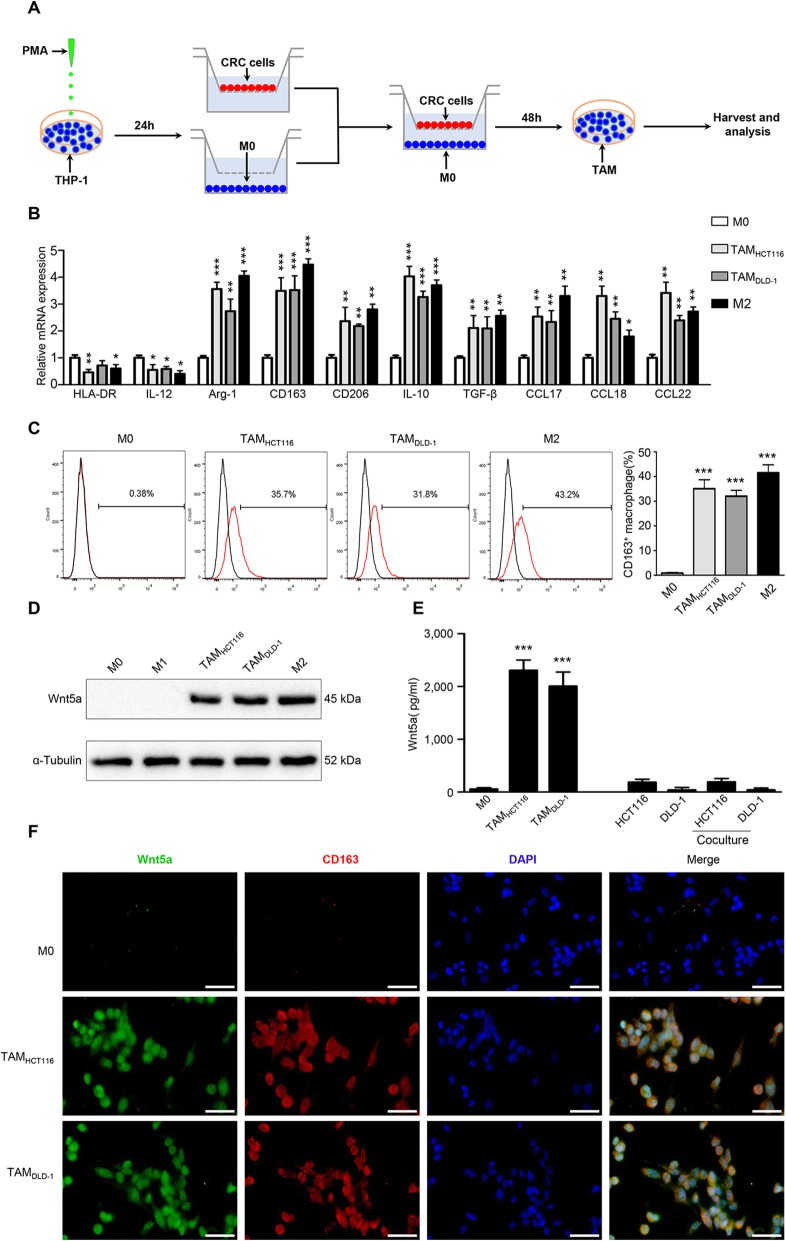
Wnt5a is mainly expressed in M2-like TAMs. **a** Flow chart of generating TAMs. **b** Relative expression of M1 markers (HLA-DR, IL-12), M2 markers (Arg-1, CD163, CD206, IL-10, TGFβ, CCL17, CCL18, CCL22) in M0 macrophages, M2 macrophages and TAMs co-cultured with HCT116 or DLD-1 for 48 h. Error bars, SEM. **c** Flow cytometry analysis of the proportion of M2 cells in different groups of macrophages. Error bars, SEM. **d** The expression level of Wnt5a in M0, M1, M2 macrophages and TAMs co-cultured with HCT116 or DLD-1 for 48 h. **e** ELISA analysis of Wnt5a secretion level in macrophages, CRC cell lines and CRC cell lines co-cultured with macrophages. Error bars, SEM. **f** Representative immunofluorescence photographs for Wnt5a, CD163 and DAPI in different groups of macrophages. Bar = 50 μm. All experiments were performed independently at least three times. Statistical analysis was conducted using one-way ANOVA. *P < 0.05. **P < 0.01. ***P < 0.001

To validate the above clinical results, we applied an in vitro model of tumor-associated macrophages according to previous reports [28]. As shown in the flowchart (Fig. 2a), after treated with PMA for 24 h, human THP-1 monocytes were differentiated into M0 macrophages and then co-cultured with CRC cells (HCT116 or DLD-1) for 48 h to generate TAMs. TAMs exhibited higher levels of M2 markers CD163, CD206, and lower levels of M1 marker HLA-DR (Fig. 2b). Flow cytometry analysis showed that the proportion of CD163 positive cells in TAMs was around 33.6, and 43.7% in IL-4/IL-13-induced M2 macrophages (Fig. 2c). Additionally, TAMs also expressed higher levels of M2 markers IL-10, TGF-β, CCL17, CCL18 and CCL22 and lower level of M1 marker IL-12 (Fig. 2b). These results suggest that TAM produced by the in vitro model is a kind of macrophage based on M2 phenotype.

We then investigated Wnt5a expression in different phenotypes of macrophages. As shown in Fig. 2d and Fig. S2A, Wnt5a was apparently overexpressed in TAMs and M2 macrophages, while scarcely expressed in M0 and M1 macrophages. Wnt5a expression in CRC cell lines was also rare or scarce (Fig. S2B). Further ELISA analysis showed that the secretion of Wn5a in TAMs was much more than that in M0 macrophages or CRC cells (HCT116 or DLD-1) (Fig. 2e). In addition, cellular immunofluorescence confirmed that Wnt5a was mainly expressed in CD163^+^ TAMs (Fig. 2f). Together, our findings reveal that Wnt5a is primarily located in M2-like TAMs.

### Wnt5a induces M2 macrophage polarization via IL-10

**Fig. 3:**
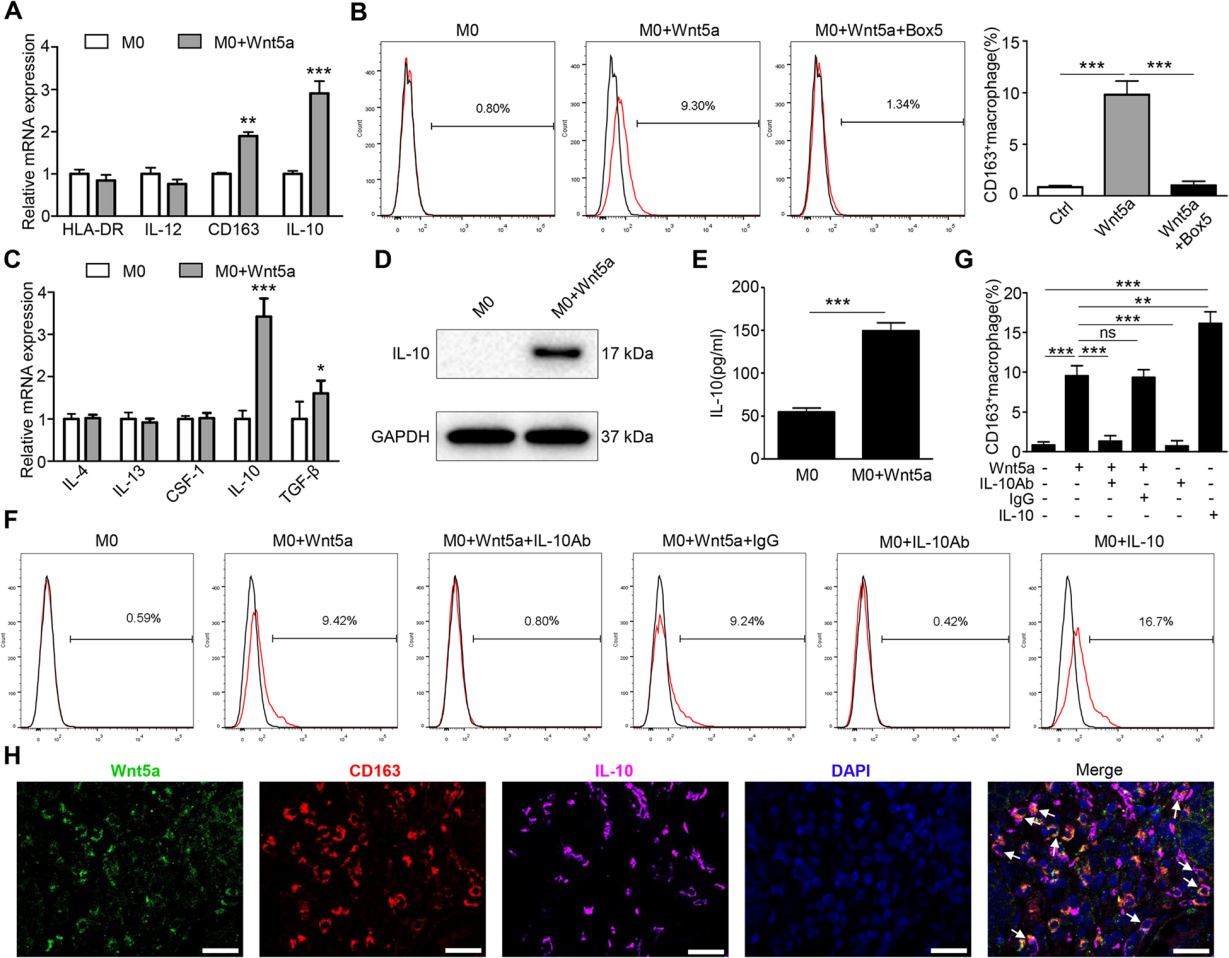
Wnt5a induces M2 macrophages polarization via IL-10. **a** RT-qPCR analysis of the relative expression levels of M1 markers (HLA-DR, IL-12) and M2 markers (CD163, IL-10) in M0 macrophages treated with Wnt5a. **b** Flow cytometry analysis showed M2 polarization (CD163^+^) in M0 macrophages treated with Wnt5a. This M2 polarization was reversed by Box 5 (Wnt5a inhibitor). **c** RT-qPCR analysis of M2 polarization-associated cytokines expression in M0 macrophages with Wnt5a treatment. **d** Western blot analyzed the protein expression of IL-10 in Wnt5a-treated M0 macrophages. **e** ELISA analysis for IL10 secretion level in M0 macrophages treated with Wnt5a. **f, g** Flow cytometry analysis of M2 cells ratio in Wnt5a-stimulated M0 macrophages with or without IL-10 antibody or IgG or recombinant IL10 protein. **h** Confocal immunofluorescence analysis showed the co-localization expression of Wnt5a, CD163 and IL-10 in CRC sample. Bar = 25 μm. All experiments were performed independently at least three times. Statistical analysis was conducted using one-way ANOVA. Error bars, SEM. ns, not significant. *P < 0.05. **P < 0.01. ***P < 0.001

Based on the above results and previous research, we assumed that Wnt5a was an important factor affecting M2 polarization. To assess the role of Wnt5a in macrophage polarization, we first treated M0 macrophages with 500 ng/ml Wnt5a for 3 days. RT-qPCR analysis showed that the expression of CD163 and IL-10 were significantly up-regulated in Wnt5a-stimulated M0 macrophages compared to untreated M0 macrophages (Fig. 3a). Subsequently, we tested the percentage of CD163^+^ cells by flow cytometry in M0 macrophages treated with Wnt5a. We found nearly 9.8% CD163^+^ macrophages in Wnt5a-stimulated M0 macrophages, which was a 10-fold increase in M2 cells (Fig. 3b). At the same time, addition of Box 5 (a specific inhibitor of Wnt5a) completely reversed the pro-M2 function of Wnt5a (Fig. 3b).

A variety of cytokines are closely related to M2 macrophage polarization [13]. Therefore, we screened several M2 polarization-associated cytokines. As shown in Fig. 3c, RT-qPCR results showed that the expression of IL-10 was significantly increased after Wnt5a stimulation. Western blot and ELISA revealed that IL-10 protein expression and secretion level were both significantly elevated (Fig. 3d and e). Wnt5a-induced M2 polarization was almost completely suppressed by IL-10 antibody (Fig. 3f and g). Additionally, confocal immunofluorescence microscopy showed that Wnt5a, CD163 and IL-10 were frequently co-localized in CRC samples (Fig. 3h). Taken together, these results demonstrate that Wnt5a induces M2 macrophage polarization via IL-10.

### CaKMII-ERK1/2-STAT3 pathway is required for Wnt5a-induced IL-10 expression in macrophages

**Fig. 4:**
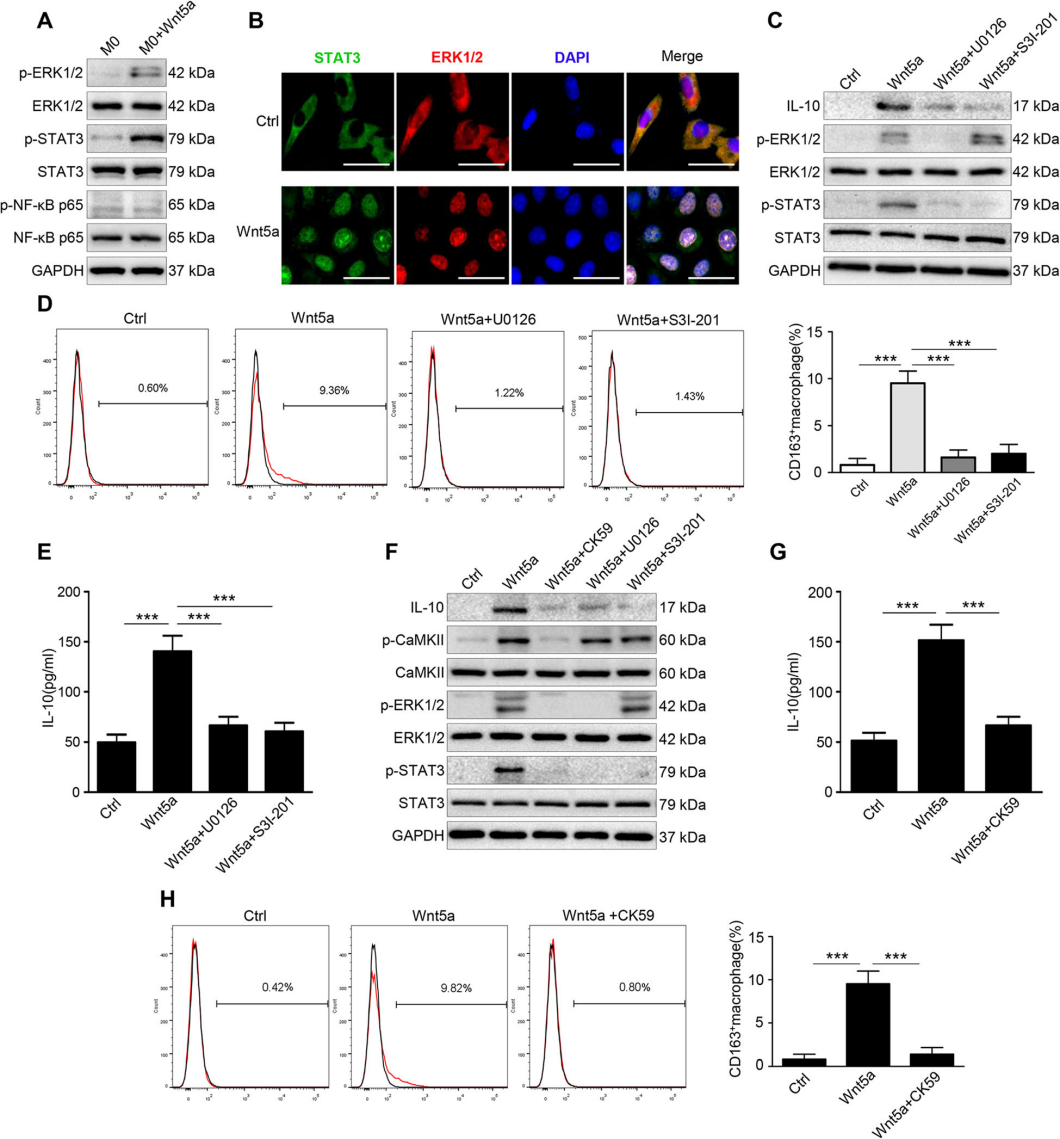
Wnt5a up-regulates IL-10 expression by activating CaKMII-ERK1/2-STAT3 pathway. **a** Western blot analysis of p-ERK1/2, p-STAT3 and p-NF-κB p65 expression in M0 macrophages with or without Wnt5a. **b** Immunofluorescence staining for ERK1/2 and STAT3 in M0 macrophages with or without Wnt5a. Bar = 25 μm. **c** Western blot analysis of IL-10, p-ERK1/2 and p-STAT3 expression in Wnt5a-treated M0 macrophages with or without U0126 or S3I-201. **d** Flow cytometry analysis of Wnt5a-treated M0 macrophages with or without U0126 or S3I-201. Error bars, SEM. **e** ELISA analysis of IL-10 secretion level in Wnt5a-treated M0 macrophages with or without U0126 or S3I-201. Error bars, SEM. **f** Western blot analysis of Wnt5a-treated M0 macrophages with or without CK59 or U0126 or S3I-201. **g** ELISA analysis of IL-10 secretion level in Wnt5a-treated M0 macrophages with or without CK59. Error bars, SEM. **h** Flow cytometry analysis of Wnt5a-treated M0 macrophages with or without CK59. Error bars, SEM. All experiments were performed independently at least three times. Statistical analysis was conducted using one-way ANOVA. ***P < 0.001

To determine the molecular mechanism by which Wnt5a up-regulated IL-10, we looked at ERK1/2, STAT3 and NF-κB signaling pathways, of which all had been reported to be involved in the regulation of IL-10 expression and macrophage polarization [25, 29, 30]. Western blot was carried out to estimate the phosphorylation status of ERK1/2, STAT3, and NF-κB in Wnt5a-treated macrophages. As shown in Fig. 4a, phosphorylation levels of ERK1/2 (p-ERK1/2) and STAT3 (p-STAT3) significantly increased after Wnt5a treatment, whereas there was no significant change in the phosphorylation level of p65 (an important component of NF-κB). Immunofluorescence analysis showed that both ERK1/2 and STAT3 translocated to the nucleus of Wnt5a-stimulated macrophages (Fig. 4b). To explore whether IL-10 were regulated by ERK1/2 and STAT3 pathway, U0126 (specific small-molecule inhibitor of p-ERK1/2) and S3I-201 (specific small-molecule inhibitor of p-STAT3) were used. Results suggested that blocking p-ERK1/2 with U0126 neutralized IL-10 induction by Wnt5a, as was the case with blocking p-STAT3 with S3I-201 (Fig. 4c). Moreover, U0126 not only suppressed p-ERK1/2 but also p-STAT3 expression, and S3I-201 hardly affected the expression of p-ERK1/2 (Fig. 4c). These results reveal that Wnt5a affects IL-10 expression through the ERK1/2 and STAT3 pathways, and STAT3 is phosphorylated by ERK1/2. In addition, U0126 or S3I-201 significantly reduced the ratio of the M2 cells in Wnt5a-treated macrophages (Fig. 4d). Consistently, the secretion level of IL-10 induced by Wnt5a is mostly abolished by U0126 or S3I-201(Fig. 4e).

Ca^2+^/CaMKII pathway is one of the important non-canonical pathways of Wnt5a signaling, which may further activate the downstream pathway of ERK1/2 [17, 31]. Hence, we tested the expression level of p-CaMKII in Wnt5a-stimulated macrophages. We found that Ca^2+^/CaMKII pathway was activated by Wnt5a (Fig. S2C). To investigate whether Wnt5a activated ERK1/2-STAT3 pathway through Ca^2+^/CaMKII pathway, western blot was carried out to test p-ERK1/2 and p-STAT3 expression in Wnt5a-treated macrophages with or without CK59 (p-CaMKII inhibitor) or U0126 or S3I-201. Results showed that Wnt5a-induced up-regulation of p-ERK1/2 and p-STAT3 expression were entirely abolished by CK59, while p-CaMKII level was not affected by U0126 or S3I-201 (Fig. 4f). Meanwhile, CK59, U0126 and S3I-201 inhibited IL-10 protein expression (Fig. 4f). ELISA and flow cytometry analyses suggested that CK59 almost completely repressed IL-10 secretion level and the pro-M2 function of Wnt5a (Fig. 4g and h). Collectively, the above results demonstrate that Wnt5a-CaMKII-ERK1/2-STAT3 pathway is crucial for IL-10-mediated M2 polarization.

### Wnt5a indirectly promotes tumor proliferation, migration and invasion through M2 macrophages

To evaluate the role of Wnt5a in CRC cells, sh-Wnt5a or sh-NC was transfected into SW480 cells and recombinant Wnt5a protein was given to treat HCT116 or DLD-1 cells directly. The transfection efficiency of sh-Wnt5a or sh-NC was measured by RT-qPCR (Fig. S3A). The CCK-8 and colony formation assay analyses showed that tumor cell proliferation was not affected by knockdown of Wnt5a (Fig. S3B and C), as well as tumor cell migration (Fig. S3D). Similarly, Wnt5a hardly affected the proliferation and migration capability of HCT116 or DLD-1 cells (Fig. S3E-J). These results indicate that Wnt5a does not directly influence the malignant biological behavior of CRC cells.

**Fig. 5:**
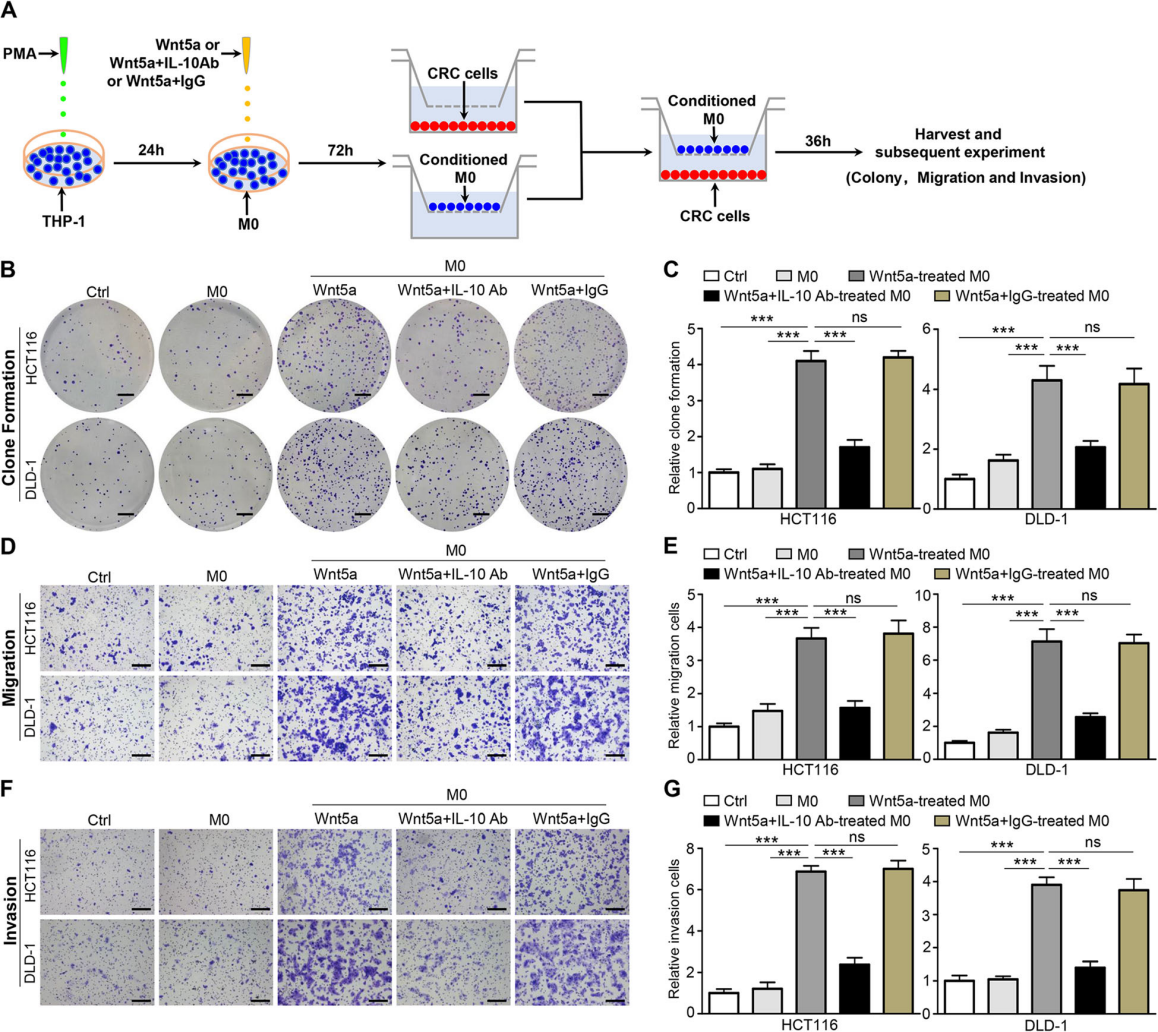
Wnt5a indirectly promotes tumor proliferation, migration and invasion through M2 macrophages. **a** Flow chart of CRC cells co-cultured with M0 macrophages treated with Wnt5a or Wnt5a + IL-10 antibody or Wnt5a + IgG. **b, c** Representative photographs and quantifications of clone formation assay in HCT116 and DLD-1 cells co-cultured with conditioned M0 macrophages. Bar = 0.5 cm. **d, e** Transwell migration assay of HCT116 and DLD-1 cells co-cultured with conditioned M0 macrophages. Bar = 200 μm. **f, g** Transwell invasion assay of HCT116 and DLD-1 cells co-cultured with conditioned M0 macrophages. Bar = 200 μm. All experiments were performed in triplicate. Statistical analysis was conducted using one-way ANOVA. Error bars, SEM. ns, not significant. ***P < 0.001

However, it is unclear whether Wnt5a affects the biological behavior of tumor cells through the crosstalk between TAMs and cancer cells. To clarify the functions of Wnt5a-induced macrophages in CRC in vitro, M0 macrophages were first treated with Wnt5a with or without IL-10 antibody or IgG for 72 h, and then co-cultured with HCT116 or DLD-1 cells for another 36 h, which were harvested for subsequent experiments (Fig. 5a). Clonogenic assay showed that M0 macrophages treated by Wnt5a obviously improved the clonogenic survival of HCT116 cells, as well as DLD-1 cells (Fig. 5b and c). After addition of IL-10 antibody antagonized the pro-M2 function of Wnt5a, this effect was reversed (Fig. 5b and c). The migration of cancer cells co-cultured with Wnt5a-induced M2 macrophages was significantly enhanced, which was reversed by IL-10 neutralizing antibody (Fig. 5d and e). In the transwell invasion assay, the invasive ability of cancer cells co-cultured with Wnt5a-induced M2 macrophages was also enhanced, and this enhancement was suppressed by IL-10 neutralizing antibody (Fig. 5f and g). Taken together, these data reveal that M2 macrophages are critical for the cancer-promoting functions of Wnt5a.

### TAMs depend on Wnt5a for their cancer-promoting roles in vitro

**Fig. 6:**
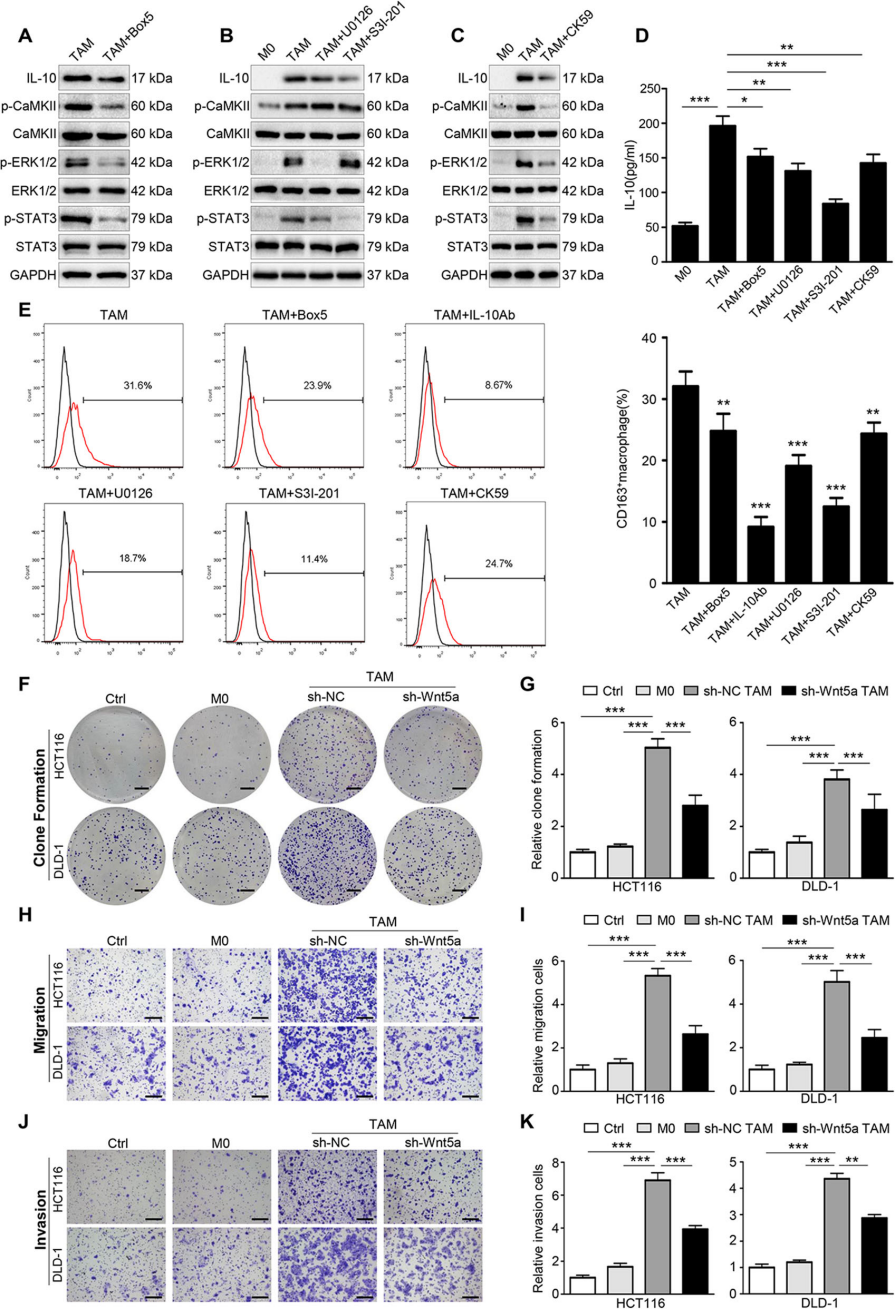
Wnt5a is crucial for the pro-tumor functions of TAMs. **a** Western blot analysis of TAMs with or without Box 5. **b** Western blot analysis of TAMs with or without U0126 or S3I-201. **c** Western blot analysis of TAMs with or without CK59. **d** ELISA detected the secretion of IL-10 in TAMs treated with inhibitors. Error bars, SEM. **e** Flow cytometry analysis of TAMs with or without inhibitors. Error bars, SEM. **f, g** Representative photographs and quantification of clone formation assay in HCT116 and DLD-1 cells co-cultured with M0 macrophages and TAMs transfected with sh-Wnt5a or sh-NC. Bar = 0.5 cm. Error bars, SEM. **h, i** Transwell migration assay of HCT116 and DLD-1 cells co-cultured with conditioned macrophages. Error bars, SEM. Bar = 200 μm. **j, k** Transwell invasion assay of HCT116 and DLD-1 cells co-cultured with conditioned macrophages. Error bars, SEM. Bar = 200 μm. All experiments were performed in triplicate. Statistical analysis was conducted using one-way ANOVA. *P < 0.05. **P < 0.01. ***P < 0.001

We further explored the role of Wnt5a in TAMs. After being treated with Box 5 (Wnt5a inhibitor), the expression level of IL-10, p-CaMKII, p-ERK1/2 and p-STAT3 were all down-regulated in TAMs (Fig. 6a). Subsequently, we verified whether the CaKMII-ERK1/2-STAT3 regulatory pathway was also present in TAMs. As shown in Fig. 6b, U0126 suppressed the level of p-STAT3 and IL-10, whereas S3I-201 only reduced the expression of IL-10 without affecting p-ERK1/2 expression. IL-10, p-ERK1/2 and p-STAT3 expression were simultaneously repressed by CK59 (Fig. 6c), while the level of p-CaMKII was not affected by U0126 or S3I-201 (Fig. 6b). The secretion level of IL-10 in TAMs was also reduced under those inhibitors’ treatment (Fig. 6d), as well as the proportion of M2 cells (Fig. 6e). These results indicate that Wnt5a is involved in the regulation of M2-like TAM polarization through CaKMII-ERK1/2-STAT3 pathway-mediated IL-10 secretion.

We then investigated the effect of Wnt5a^+^ TAMs on CRC cells. Lentiviral vectors expressing sh-Wnt5a or sh-NC were transfected into TAMs for further functional analysis in vitro. The transfection efficiency was measured by RT-qPCR and western blot (Fig. S4 A and B). Clone formation assay showed that the proliferation capacity of CRC cells co-cultured with TAMs was significantly stronger than that co-cultured with sh-Wnt5a TAMs (Fig. 6f and g). Similarly, transwell assay suggested that the migration and invasion capacity of CRC cells were dramatically enhanced by TAMs, and this enhancement was obviously attenuated after knockdown of Wnt5a in TAMs (Fig. 6h-k). Together, our findings demonstrate that Wnt5a is crucial for TAM-mediated migration and invasion of cancer cells.

### Silencing Wnt5a inhibits the pro-tumor effect of TAMs in vivo

**Fig. 7:**
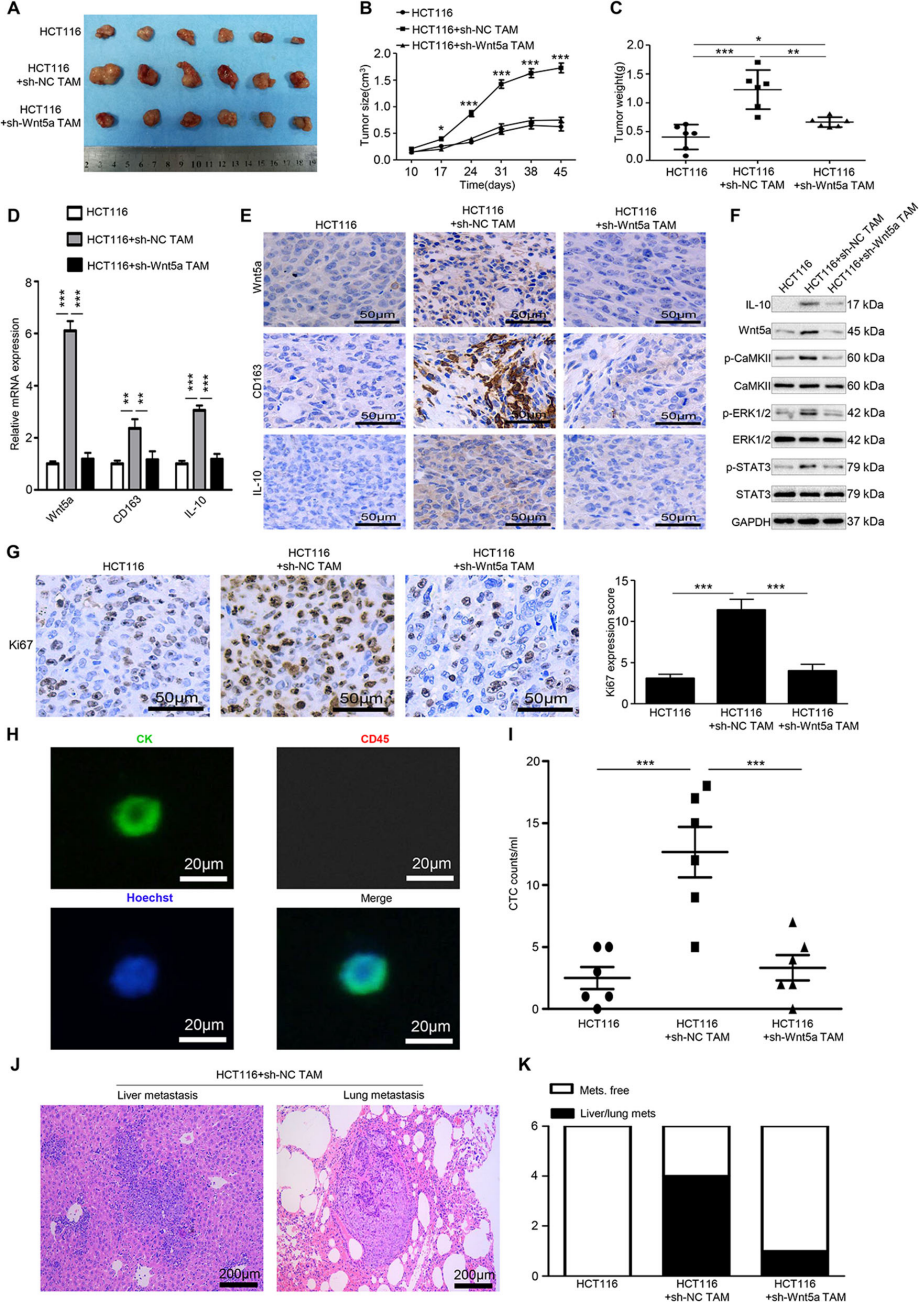
Silencing Wnt5a suppresses M2-like TAM-mediated tumor growth and metastasis in vivo. **a-c** The morphological characteristics of tumor xenograft, tumor size and tumor weight in the HCT116 alone, HCT116 + sh-NC TAM and HCT116 + sh-Wnt5a TAM groups. Error bars, SEM. **d** Relative mRNA expression of Wnt5a, CD163 and IL-10 of tumors in different groups. Error bars, SEM. **e** IHC analyzed the expression of Wnt5a, CD163 and IL-10 protein of tumors in different groups. Bar = 50 μm. **f** Western blot analysis of Wnt5a, IL-10, p-CaMKII, p-ERK1/2, and p-STAT3 expression in different groups. **g** IHC analyzed Ki67 expression of tumors in different groups. Bar = 50 μm. Error bars, SEM. **h** Representative CTC images of immunofluorescence. Bar = 20 μm. **i** Quantification of CTCs captured from the blood of mice. Error bars, SEM. **j** Representative H&E stained sections of metastatic nodules in liver and lung tissue from mice in the HCT116+ sh-NC TAM group were shown. Bar = 200 μm. **k** Quantification of mice with metastasis lesions in different groups. Statistical analysis was conducted using one-way ANOVA. *P < 0.05. **P < 0.01. ***P < 0.001

To confirm the above in vitro results, an in vivo xenograft model was used. HCT116 cells alone, TAMs alone, HCT116 + sh-NC TAMs and HCT116 + sh-Wnt5a TAMs were injected subcutaneously into the right flank of female nude mice. After 45 days, tumors in the HCT116 + sh-NC TAMs group were significantly larger and heavier than those in the HCT116 + sh-Wnt5a TAMs and HCT116 group (Fig. 7a-c). No tumors were produced in the TAMs group (data not shown). Subsequently, RT-qPCR (Fig. 7d) and immunohistochemical staining (Fig. 7e) showed that in the group injected with HCT116 + sh-Wnt5a TAMs, Wnt5a, CD163 and IL-10 expression levels were significantly lower compared with the HCT116 + sh-NC TAMs group. The protein levels of Wnt5a, IL-10, p-CaMKII, p-ERK1/2, and p-STAT3 were significantly higher in the HCT116 + sh-NC TAMs group compared with the other two groups (Fig. 7f). Additionally, Ki67 staining, an indicator of tumor proliferation, also decreased in the HCT116 + sh-Wnt5a TAMs group compared with HCT116 + sh-NC TAMs group (Fig. 7g). These data indicate that sh-Wnt5a TAMs have a much lower tumor support capacity compared with sh-NC TAMs. It is essential for cancer cells to enter the blood vessel to become CTCs for distant metastasis. We then tested the presence of CTCs in peripheral blood of the mice. Representative CTC images were presented in Fig. 7h. Further counting analysis showed significant more CTCs were detected in the HCT116 + sh-NC TAM group compared with the other two groups (Fig. 7i). Liver/lung metastatic lesions were found in one out of six mice injected with HCT116 + sh-Wnt5a TAMs, none in mice injected with only HCT116 cells, while four in mice injected with HCT116 + sh-NC TAM (Fig. 7j and kj and k). Collectively, these results demonstrate that TAMs enhance the growth and metastasis of CRC, whereas Wnt5a knockdown impairs the cancer-promoting functions of TAMs in vivo.

## Discussion

In this study, we firstly found that high Wnt5a^+^CD68^+^/CD68^+^ TAMs ratio was significantly associated with clinicopathologic characteristics and poor prognosis in CRC patients. Further investigation confirmed that Wnt5a was involved in M2 polarization of TAMs by regulating CaMKII-ERK1/2-STAT3 pathway-mediated IL-10 secretion, thereby promoting CRC progression.

Clinically, elevated Wnt5a expression in CRC was found to be mainly localized in TAMs. High Wnt5a^+^ TAMs/TAMs (Wnt5a^+^CD68^+^/CD68^+^) ratio was significantly correlated with LVI, TI, LNM, TNM stage and poor prognosis in CRC patients. Previously, the association of TAMs with cancer prognosis had been extensively studied. Some studies showed that TAMs infiltration was a poor prognostic factor [32–34], while others reported that TAMs infiltration was associated with better prognosis [35–37]. The discrepancy was especially pronounced in CRC [32, 35, 37]. We noticed that CD68 was used in these studies to identify TAMs, which was generally considered to be a pan-macrophage marker, making this protein unspecific for distinguishing TAM subtypes and their different roles in CRC progression. On the other hand, TAM subtypes were found to be a better choice for predicting cancer prognosis. Numerous studies had shown that high CD163^+^ TAM subtype infiltration was significantly associated with poor clinical outcomes in numerous solid tumors [8, 38, 39]. Cortes et al. revealed that ZEB1^+^ TAM subtype was correlated with tumor growth, chemotherapy resistance and poor survival in ovarian carcinoma [40]. In this work, we confirmed that Wnt5a^+^ TAM subtype was a preferable indicator of CRC prognosis to TAM. Therefore, Wnt5a^+^ TAM subtype could serve as a poor prognostic maker for evaluating prognosis in CRC patents.

Functional polarization of TAMs to M1 or M2 represents a key mechanism that controls their functions, switching roles between tumor suppression and promotion [10, 41]. Multiple studies tended to M2 TAMs, which were closely related to poor prognosis in CRC [8, 42]. We found that Wnt5a was co-localized with CD163 (M2 marker), rather than HLA-DR (M1 marker). We also found that Wnt5a was involved in inducing macrophage polarization to M2 phenotype. These results indicate that Wnt5a^+^ TAM is an M2-like TAM subtype. Further in vitro and in vivo experiments confirmed that Wnt5a^+^ TAMs promoted CRC cells growth, migration, invasion and metastasis. Knocking down Wnt5a mostly impaired the pro-tumor effect of TAMs, which revealed that TAMs depended on Wnt5a for their cancer-promoting roles. Intriguingly, knocking down Wnt5a did not alter the proliferation and migration capacity of SW480, and direct stimulation with recombinant Wnt5a protein also hardly affected the proliferation and migration ability of HCT116 or DLD-1 cells. These results reveal that Wnt5a does not directly influence CRC cells, but indirectly facilitates cancer progression by regulating TAM M2 polarization. In contrast, a few studies showed that Wnt5a had no or weak expression in colorectal cancer and mainly played a role in tumor suppression [43, 44]. Their research focused on the direct effects of Wnt5a on cancer cells, while ours concentrated on the regulatory role of Wnt5a in TAMs. In fact, Smith and colleagues reported a long time ago that Wnt5a was mainly expressed in TAMs in CRC [24], which is consistent with our findings. In addition, Nitzki et al. also found that Wnt5a was mainly derived from the tumor stroma, especially from TAMs in basal cell carcinoma [23]. Therefore, we inferred that Wnt5a was more likely to play a major role in altering the tumor microenvironment, regulating M2-like TAM polarization, inhibiting tumor immune response, and thus contributing to tumor invasion and metastasis. These data demonstrate that M2-like TAM is an important mediator of Wnt5a to promote CRC progression.

M2 polarization of macrophages is well known to be driven by multiple signaling pathways and cytokines including IL-4, IL-13 and IL-10 [13, 45]. In terms of cytokines, here we demonstrated that Wnt5a stimulated macrophages to produce IL-10, which in turn induced M2 polarization of macrophages, finally facilitating tumor growth, invasion and metastasis. This observation revealed that the protumorigenic effects of Wnt5a were mediated by IL-10-induced M2-like TAM polarization. Similarly, this way of regulating IL-10 expression was also observed in the study by Lee and colleagues. They stated that BMP-6 (bone morphogenetic proteins-6) stimulated TAMs to produce IL-10, which then induced M2 polarization of TAMs to mediate the pro-tumor effect of BMP-6 [30]. Mechanistically, we demonstrated that CaMKII-ERK1/2-STAT3 pathway was essential for IL-10 production induced by Wnt5a. It had been confirmed that STAT3 bound directly to the − 588 to − 800 bp region of IL-10 promoter to promote IL-10 transcription [30]. Ma et al. found that dysregulation of CaMKII in intestinal epithelial cells regulated colitis-associated colorectal carcinogenesis by enhancing STAT3 activation [46]. Unudurthi et al. showed that CaMKII was aberrantly activated in cardiac hypertrophy and heart failure, leading to degradation of βIV-spectrin, resulting in an increase of free STAT3 level [47]. However, it was unclear in their work whether STAT3 was phosphorylated either by CaMKII or indirectly through another kinase. Zhang and colleagues reported that ERK1/2 activated the STAT3 pathway in the regulation of M2-like TAMs polarization [29]. We also identified that CaMKII indirectly promoted STAT3 phosphorylation through the ERK1/2 pathway. On the whole, Wnt5a-CaMKII-ERK1/2-STAT3 pathway plays an important role in M2 polarization. Their specific regulatory mechanisms are still unclear, especially the regulation of STAT3 by ERK1/2. Further investigation is necessary to clarify the mechanism by which ERK1/2 regulates the STAT3 pathway.

In this work, we demonstrated that TAMs secreted both Wnt5a and IL-10, which formed a Wnt5a/IL-10 autocrine loop, resulting in M2 polarization of TAMs. Previously, multiple studies showed that Wnt5a was induced by inflammatory factors [25, 48]. Inflammation microenvironment is a recognized hallmark of CRC [49], and CRC cells could also secrete a variety of inflammatory factors to promote tumor invasion and metastasis [50, 51]. Therefore, the inflammatory factors in tumor microenvironment may play an important role in Wnt5a induction. Further exploration is required to elucidate the mechanism by which Wnt5a expression is up-regulated in TAMs.

M2 polarization of TAMs is a multi-factor, multi-step and complex pathological process in the tumor microenvironment [13]. Recently, we found that IL-4 secreted by colorectal cancer cells affected M2 polarization of TAM [9]. Moreover, factors such as GM-CSF [12] and exosomes [52] derived from cancer cells are involved in inducing macrophages M2 polarization. Therefore, many factors derived from cancer cells or tumor microenvironment could affect M2 polarization of macrophages during colorectal cancer progression. M2 macrophages are generally considered to inhibit inflammatory responses and promote tumor immune escape during tumor progression [6]. In this study, we demonstrated that Wnt5a induced M2 macrophage polarization via IL-10. In contrast, Pereira et al. showed that Wnt5a was induced by LPS/IFN-γ and suppressed by IL-10 in macrophages [53]. Their research was mainly based on changes in mRNA expression level of Wnt5a [53]. However, Leandersson and colleagues showed that Wnt5a was regulated at the post-transcriptional level, and the mRNA expression level of Wnt5a is not closely related to its protein level, indicating that Wnt5a mRNA expression could not reflect its protein level [54]. In addition, Pereira’s study only emphasized the upregulation of Wnt5a expression in macrophages derived from sepsis patients, and did not further explore the effect of Wnt5a on macrophage differentiation [53]. However, Bergenfelz et al. had confirmed that Wnt5a induced LPS/IFN-γ-activated M1 macrophages to dedifferentiate into tolerogenic phenotype of macrophages via IL-10 in sepsis [25]. Li and colleagues also found that Wnt5a inhibited inflammation-driven intervertebral disc degeneration through negative feedback regulation of NF-kB [55]. Therefore, Wnt5a might be an important mediator to suppress inflammatory response of macrophages in a negative feedback manner.

## Conclusion

In summary, our results revealed that, as an M2-like TAM subtype, high Wnt5a^+^ TAMs/CD68^+^ TAMs ratio was significantly associated with poor prognosis in CRC patients. Mechanistically, Wnt5a induced M2 polarization of TAMs by regulating CaMKII-ERK1/2-STAT3 pathway-mediated IL-10 secretion, thereby promoting tumor growth, invasion and metastasis of CRC. Our study highlighted that Wnt5a^+^ TAM or Wnt5a/CaMKII/ERK/STAT3/IL-10 axis might be a novel potential immunotherapy target for combating CRC.

## Supplementary information

**Additional file 1: Table S1.** Correlation between the density of macrophages and clinicopathologic parameters (*n* = 63). **Table S2.** The sequences of the primers for RT-qPCR. **Figure S1.** Wnt5a^+^ TAM is significantly associated with prognosis in CRC patients. (a) Representative IHC staining of Wnt5a in CRC sample. Bar = 100 μm. (b) Wnt5a^+^ TAMs expression was significantly elevated in primary CRC tissues compared with normal colorectal tissues. Error bars, SEM. Statistical analysis was conducted using one-way ANOVA. (c) Correlation analysis between Wnt5a^+^ TAMs expression and RFS of CRC patients. (d) Correlation analysis between Wnt5a^+^ TAMs expression and OS of CRC patients. (e) Association of TAMs expression with RFS of CRC patients. (f) Association of TAMs expression with OS of CRC patients. ***P < 0.001. **Figure S2.** (a) Relative expression of Wnt5a mRNA in M0, M1, M2 macrophages and TAMs cocultured with HCT116 or DLD-1. Error bars, SEM. (b) Western blot analysis of Wnt5a expression in CRC cell lines and CRC cell lines co-cultured with macrophages. (c) Western blot analysis of the level of p-CaKMII in Wnt5a-treated M0 macrophages. All experiments were performed in triplicate. Statistical analysis was conducted using one-way ANOVA. ns, not significant. ***P < 0.001. **Figure S3.** Wnt5a does not directly influence the malignant biological behavior of CRC cells. (a) RT-qPCR analysis of Wnt5a expression in SW480 cells transfected with sh-Wnt5a or sh-NC. (b) CCK-8 assay analysis of cell viability in SW480 cells transfected with sh-Wnt5a or sh-NC. (c) Representative photographs and quantification of clone formation assay in SW480 cells transfected with sh-Wnt5a or sh-NC. Bar = 0.5 cm. (d) Transwell migration assay analysis of SW480 cells transfected with sh-Wnt5a or sh-NC. Bar = 200 μm. (e, f) CCK-8 assay analysis of cell viability in HCT116 or DLD-1 cells treated with Wnt5a. (g, h) Representative photographs and quantification of clone formation assay in HCT116 or DLD-1 cells treated with Wnt5a. Bar = 0.5 cm. (i, j) Transwell migration assay analysis of HCT116 or DLD-1 cells treated with Wnt5a. Bar = 200 μm. Error bars, SEM. All experiments were performed in triplicate. Statistical analysis was conducted using Student’s t test. ns, not significant. *P < 0.05. ***P < 0.001. **Figure S4.** (a) RT-qPCR analysis of Wnt5a expression in M0 macrophages, TAMs and TAMs transfected with sh-Wnt5a or sh-NC. Error bars, SEM. (b) Western blot analysis of Wnt5a expression in M0 macrophages, TAMs and TAMs transfected with sh-Wnt5a or sh-NC. All experiments were performed in triplicate. Statistical analysis was conducted using one-way ANOVA. ns, not significant. ***P < 0.001.

## Data Availability

The original data will be transferred and shared following the instruction by the Editorial Committee.

## Abbreviations

TME: Tumor microenvironment
TAMs: Tumor-associated macrophages
EMT: Epithelial-mesenchymal transition
CRC: Colorectal cancer
CTC: Circulating tumor cell
Wnt5a: Wingless-type MMTV integration site family, member 5a
IL-10: Interleukin-10
RFS: Recurrence-free survival
OS: Overall survival
ERK: Extracellular regulated protein kinases
STAT3: Signal transducer and activator of transcription 3
CaMKII: Calmodulin-dependent protein kinase II
