# Supplementary material for: Wnt5a-induced M2 polarization of tumor-associated macrophages via IL-10 promotes colorectal cancer progression
Source: Cell Commun Signal. 2020 Mar 30;18:51. doi: 10.1186/s12964-020-00557-2 (PMC7106599; doi:10.1186/s12964-020-00557-2)
Supplement: Supplementary file 1 — Additional file 1: Table S1. Correlation between the density of macrophages and clinicopathologic parameters (n = 63). Table S2. The sequences of the primers for RT-qPCR. Figure S1. Wnt5a+ TAM is significantly associated with prognosis in CRC patients. (a) Representative IHC staining of Wnt5a in CRC sample. Bar = 100 μm. (b) Wnt5a+ TAMs expression was significantly elevated in primary CRC tissues compared with normal colorectal tissues. Error bars, SEM. Statistical analysis was conducted using one-way ANOVA. (c) Correlation analysis between Wnt5a+ TAMs expression and RFS of CRC patients. (d) Correlation analysis between Wnt5a+ TAMs expression and OS of CRC patients. (e) Association of TAMs expression with RFS of CRC patients. (f) Association of TAMs expression with OS of CRC patients. ***P < 0.001. Figure S2. (a) Relative expression of Wnt5a mRNA in M0, M1, M2 macrophages and TAMs cocultured with HCT116 or DLD-1. Error bars, SEM. (b) Western blot analysis of Wnt5a expression in CRC cell lines and CRC cell lines co-cultured with macrophages. (c) Western blot analysis of the level of p-CaKMII in Wnt5a-treated M0 macrophages. All experiments were performed in triplicate. Statistical analysis was conducted using one-way ANOVA. ns, not significant. ***P < 0.001. Figure S3. Wnt5a does not directly influence the malignant biological behavior of CRC cells. (a) RT-qPCR analysis of Wnt5a expression in SW480 cells transfected with sh-Wnt5a or sh-NC. (b) CCK-8 assay analysis of cell viability in SW480 cells transfected with sh-Wnt5a or sh-NC. (c) Representative photographs and quantification of clone formation assay in SW480 cells transfected with sh-Wnt5a or sh-NC. Bar = 0.5 cm. (d) Transwell migration assay analysis of SW480 cells transfected with sh-Wnt5a or sh-NC. Bar = 200 μm. (e, f) CCK-8 assay analysis of cell viability in HCT116 or DLD-1 cells treated with Wnt5a. (g, h) Representative photographs and quantification of clone formation assay in HCT116 or DLD-1 cells treat [file 12964_2020_557_MOESM1_ESM.docx]

| Table S1 Correlation between the density of macrophages and clinicopathologic parameters (n = 63). | | | | | | | | |  |
| --- | --- | --- | --- | --- | --- | --- | --- | --- | --- |
| Parameters | n (%) | CD68^+^ expression (n) | | |  | Wnt5a^+^CD68^+^ expression (n) | | | |
|  |  | Low (n=32) | High (n=31) | *P* value |  | Low (n=32) | High (n=31) | *P* value | |
| Gender |  |  |  | 0.089 |  |  |  | 0.503 | |
| Male | 38 (60.32) | 16 | 22 |  |  | 18 | 20 |  | |
| Female | 25 (39.68) | 16 | 9 |  |  | 14 | 11 |  | |
| Age (years) |  |  |  | 0.167 |  |  |  | 0.712 | |
| ＜60 | 29 (46.03) | 12 | 17 |  |  | 14 | 15 |  | |
| ≥60 | 34 (53.97) | 20 | 14 |  |  | 18 | 16 |  | |
| Tumor site |  |  |  | 0.159 |  |  |  | 0.535 | |
| Colon | 35 (55.56) | 15 | 20 |  |  | 19 | 16 |  | |
| Rectum | 28 (44.44) | 17 | 11 |  |  | 13 | 15 |  | |
| Tumor size (cm) |  |  |  | 0.250 |  |  |  | 0.093 | |
| ＜5 | 41 (65.08) | 23 | 18 |  |  | 24 | 17 |  | |
| ≥5 | 22 (34.92) | 9 | 13 |  |  | 8 | 14 |  | |
| Tumor rade |  |  |  | 0.719 |  |  |  | 0.382 | |
| Poor | 38 (60.32) | 20 | 18 |  |  | 21 | 17 |  | |
| Moderate/Well | 25 (39.68) | 12 | 13 |  |  | 11 | 14 |  | |
| LVI |  |  |  | 0.163 |  |  |  | **0.016** | |
| Absence | 30 (47.62) | 18 | 12 |  |  | 20 | 10 |  | |
| Presence | 33 (52.38) | 14 | 19 |  |  | 12 | 21 |  | |
| PNI |  |  |  | 0.521 |  |  |  | 0.712 | |
| Absence | 34 (53.97) | 16 | 18 |  |  | 18 | 16 |  | |
| Presence | 29 (46.03) | 16 | 13 |  |  | 14 | 15 |  | |
| TI |  |  |  | 0.338 |  |  |  | 0.714 | |
| T1-2 | 15 (23.81) | 6 | 9 |  |  | 7 | 8 |  | |
| T3-4 | 48 (76.19) | 26 | 22 |  |  | 25 | 23 |  | |
| LNM |  |  |  | 0.101 |  |  |  | 0.527 | |
| N0-1 | 31 (49.21) | 19 | 12 |  |  | 17 | 14 |  | |
| N2-3 | 32 (50.79) | 13 | 19 |  |  | 15 | 17 |  | |
| TNM stage^#^ |  |  |  | **0.032** |  |  |  | **0.002** | |
| I/II | 33 (52.38) | 21 | 12 |  |  | 23 | 10 |  | |
| III | 30 (47.62) | 11 | 19 |  |  | 9 | 21 |  | |
| CEA (ng/ml) |  |  |  | 0.365 |  |  |  | 0.146 | |
| ＜5 | 44 (69.84) | 24 | 20 |  |  | 25 | 19 |  | |
| ≥5 | 19 (30.16) | 8 | 11 |  |  | 7 | 12 |  | |
| Notes: Bold indicates *P* < 0.05; ^#^The 7th edition of the AJCC Cancer Staging Manual. Abbreviations: LVI, lymphovascular invasion; PNI, perineural invasion; TI, tumor invasion; LNM, lymph node metastasis; TNM, tumor-node-metastasis; CEA, carcinoembryonic antigen; CD68, cluster of differentiation 68; Wnt5a, wingless-type MMTV integration site family, member 5a. | | | | | | | | |  |

| Table S2 The sequences of the primers for RT-qPCR | | |
| --- | --- | --- |
| Genes | Primer Sequence (5' to 3') | Product size (bp) |
| HLA-DR | F: TCTGGCGGCTTGAAGAATTTG | 125 |
|  | R: GGTGATCGGAGTATAGTTGGAGC |  |
| IL-12 | F: ACCTGACCCACCCAAGAACT | 131 |
|  | R: GGACCTGAACGCAGAATGTC |  |
| Arginase 1 | F: TGGACAGACTAGGAATTGGCA | 102 |
|  | R: CCAGTCCGTCAACATCAAAACT |  |
| CD163 | F: TTGTCAACTTGAGTCCCTTCAC | 127 |
|  | R: TCCCGCTACACTTGTTTTCAC |  |
| CD206 | F: GGGTTGCTATCACTCTCTATGC | 126 |
|  | R: TTTCTTGTCTGTTGCCGTAGTT |  |
| IL-10 | F: GCCAAGCCTTGTCTGAGATGATCC | 91 |
|  | R: TTCACATGCGCCTTGATGTCTGG |  |
| TGF-β | F: AAGGACCTCGGCTGGAAGTGC | 136 |
|  | R: CCGGGTTATGCTGGTTGTA |  |
| CCL17 | F: CTTCAAGGGAGCCATTCCCC | 129 |
|  | R: CTCTTGTTGTTGGGGTCCGA |  |
| CCL18 | F: CTCTGCTGCCTCGTCTATACCT | 108 |
|  | R: CTTGGTTAGGAGGATGACACCT |  |
| CCL22 | F: CCGCTCTGCAGGGTATTTGA | 111 |
|  | R: GCCCCACAGCAAGCCTATAA |  |
| Wnt5a | F: GTTTCGGCTACAGACCCAGA | 92 |
|  | R: CCCCAGTTCATTCACACCACA |  |
| IL-4 | F: ACAGCAGTTCCACAGGCACAAG | 115 |
|  | R: CGTACTCTGGTTGGCTTCCTTCAC |  |
| IL-13 | F: GGTATGGAGCATCAACCTGACAGC | 115 |
|  | R: GCAGAATCCGCTCAGCATCCTC |  |
| CSF-1 | F: AGCCAGAAGGAGGACCAGCAAG | 145 |
|  | R: ACCAGCAGGTGGAAGACAGACTC |  |
| GAPDH | F: GCACCACCAACTGCTTAGCA | 106 |
|  | R: GTCTTCTGGGTGGCAGTGATG |  |


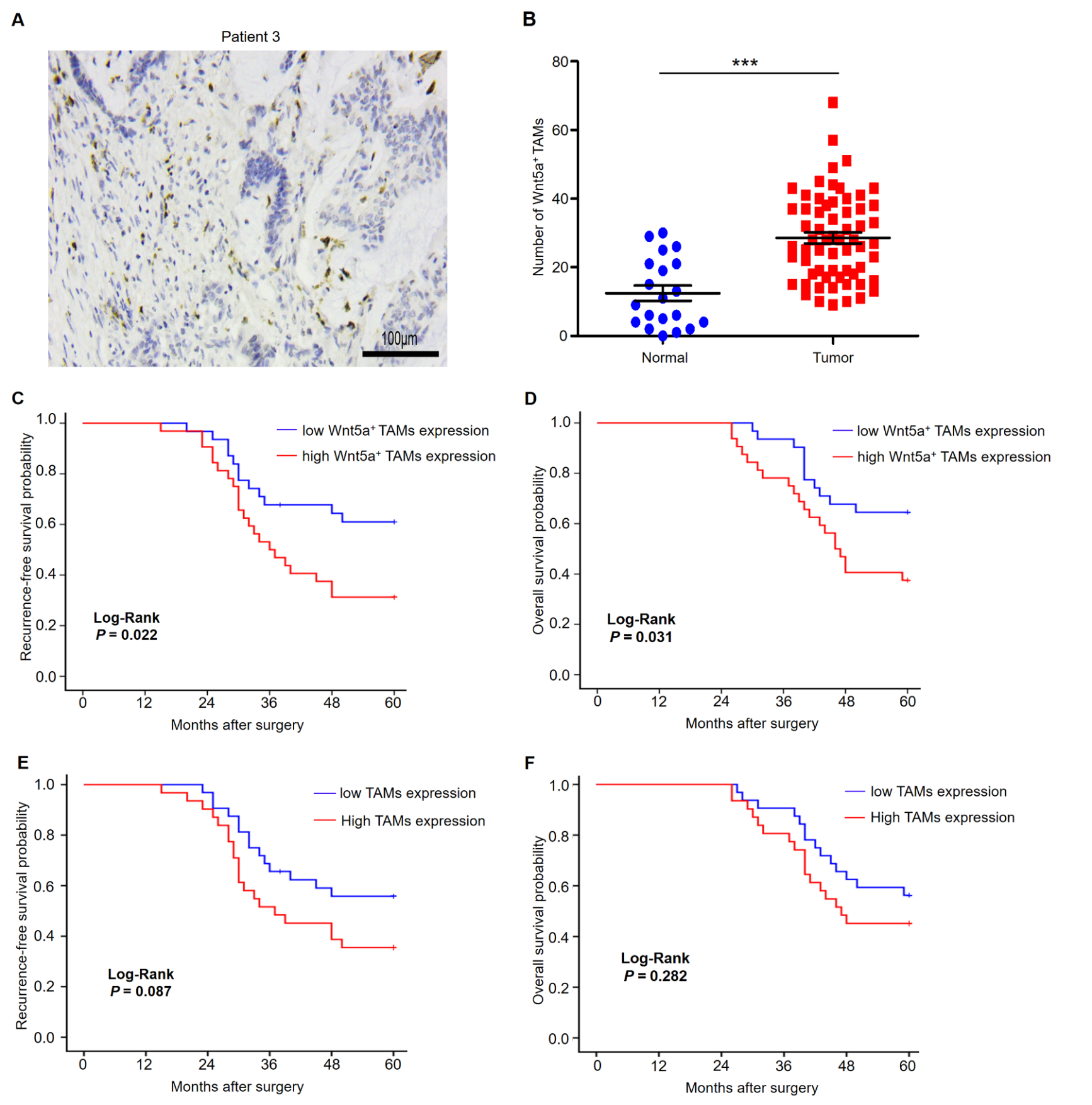


**Figure S1.** Wnt5a^+^ TAM is significantly associated with prognosis in CRC patients. (a) Representative IHC staining of Wnt5a in CRC sample. Bar = 100μm. (b) Wnt5a^+^ TAMs expression was significantly elevated in primary CRC tissues compared with normal colorectal tissues. Error bars, SEM. Statistical analysis was conducted using one-way ANOVA. (c) Correlation analysis between Wnt5a^+^ TAMs expression and RFS of CRC patients. (d) Correlation analysis between Wnt5a^+^ TAMs expression and OS of CRC patients. (e) Association of TAMs expression with RFS of CRC patients. (f) Association of TAMs expression with OS of CRC patients. ***P<0.001


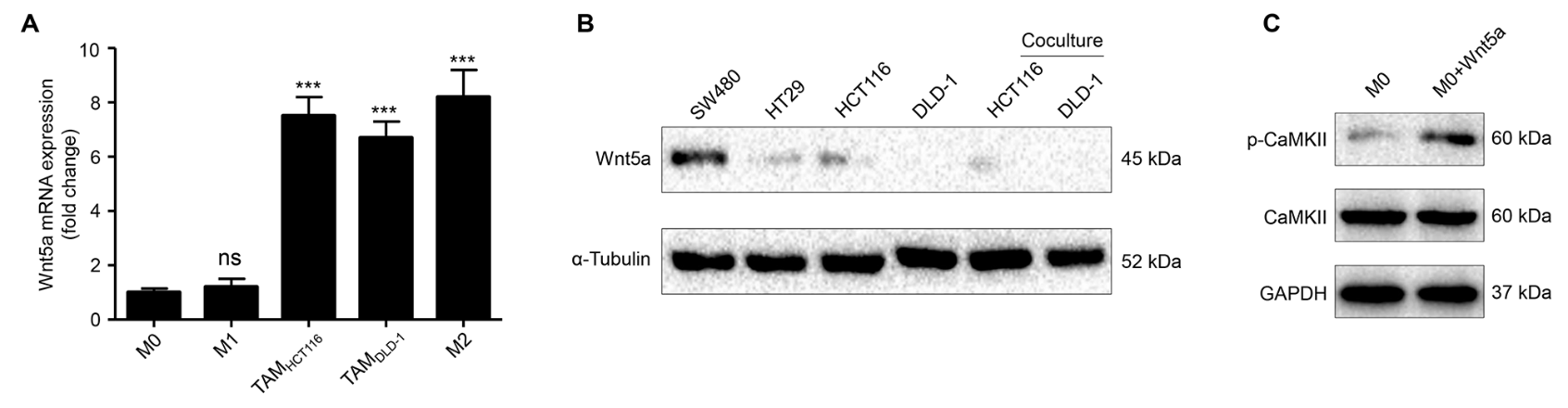


**Figure S2.** (a) Relative expression of Wnt5a mRNA in M0, M1, M2 macrophages and TAMs cocultured with HCT116 or DLD-1. Error bars, SEM. (b) Western blot analysis of Wnt5a expression in CRC cell lines and CRC cell lines co-cultured with macrophages. (c) Western blot analysis of the level of p-CaKMII in Wnt5a-treated M0 macrophages. All experiments were performed in triplicate. Statistical analysis was conducted using one-way ANOVA. ns, not significant. ***P<0.001


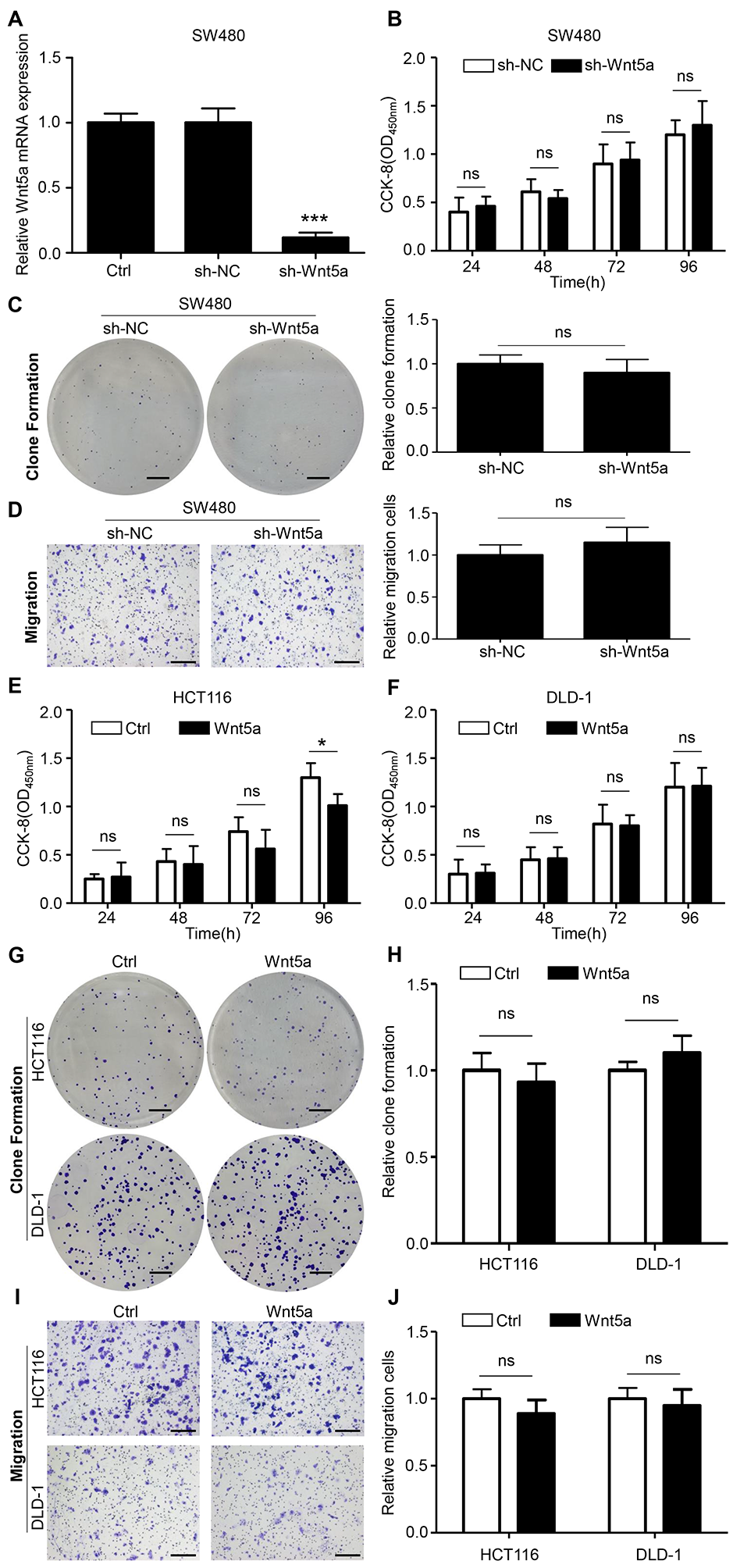


**Figure S3.** Wnt5a does not directly influence the malignant biological behavior of CRC cells. (a) RT-qPCR analysis of Wnt5a expression in SW480 cells transfected with sh-Wnt5a or sh-NC. (b) CCK-8 assay analysis of cell viability in SW480 cells transfected with sh-Wnt5a or sh-NC. (c) Representative photographs and quantification of clone formation assay in SW480 cells transfected with sh-Wnt5a or sh-NC. Bar = 0.5cm. (d) Transwell migration assay analysis of SW480 cells transfected with sh-Wnt5a or sh-NC. Bar = 200μm. (e, f) CCK-8 assay analysis of cell viability in HCT116 or DLD-1 cells treated with Wnt5a. (g, h) Representative photographs and quantification of clone formation assay in HCT116 or DLD-1 cells treated with Wnt5a. Bar = 0.5cm. (i, j) Transwell migration assay analysis of HCT116 or DLD-1 cells treated with Wnt5a. Bar = 200μm. Error bars, SEM. All experiments were performed in triplicate. Statistical analysis was conducted using Student’s t test. ns, not significant. *P<0.05. ***P<0.001


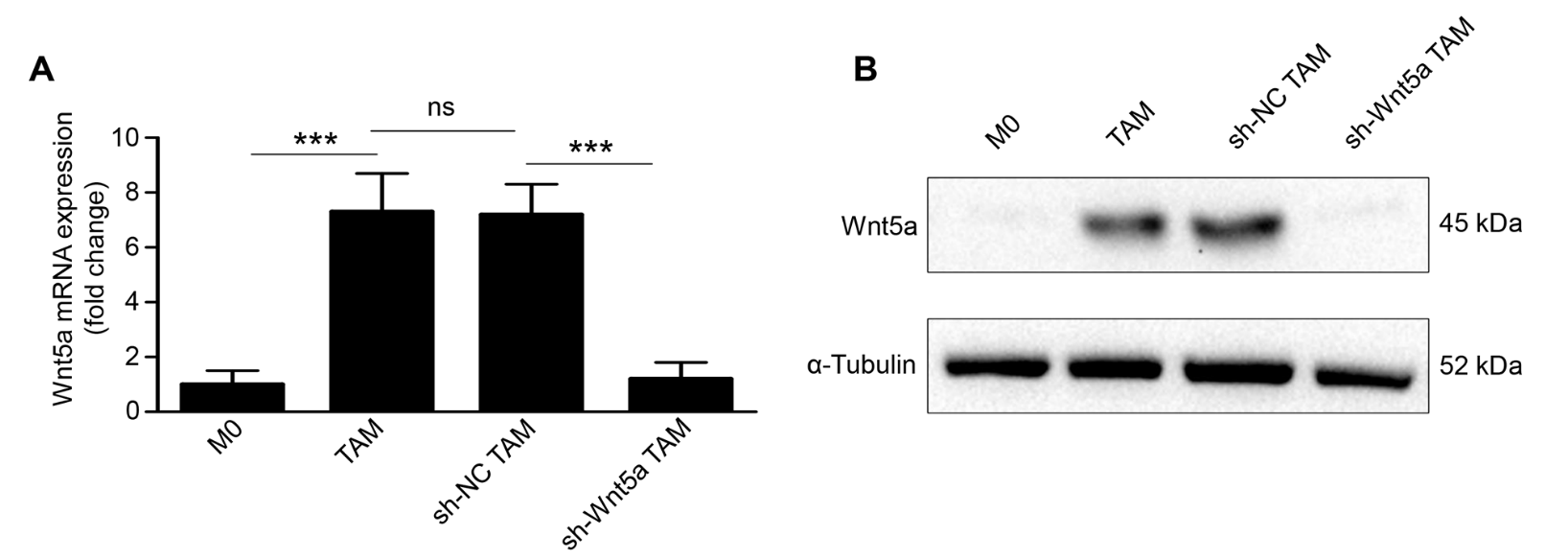


**Figure S4.** (a) RT-qPCR analysis of Wnt5a expression in M0 macrophages, TAMs and TAMs transfected with sh-Wnt5a or sh-NC. Error bars, SEM. (b) Western blot analysis of Wnt5a expression in M0 macrophages, TAMs and TAMs transfected with sh-Wnt5a or sh-NC. All experiments were performed in triplicate. Statistical analysis was conducted using one-way ANOVA. ns, not significant. ***P<0.001
